# A hybrid machine learning feature selection model—HMLFSM to enhance gene classification applied to multiple colon cancers dataset

**DOI:** 10.1371/journal.pone.0286791

**Published:** 2023-11-02

**Authors:** Murad Al-Rajab, Joan Lu, Qiang Xu, Mohamed Kentour, Ahlam Sawsa, Emad Shuweikeh, Mike Joy, Ramesh Arasaradnam

**Affiliations:** 1 College of Engineering, Abu Dhabi University, Abu Dhabi, United Arab Emirates; 2 School of Computing and Engineering, University of Huddersfield, Huddersfield, United Kingdom; 3 Bradford Teaching Hospitals NHS Foundation Trust, Bradford, United Kingdom; 4 University of Warwick, Coventry, United Kingdom; 5 University Hospital Coventry & Warwickshire, Coventry, United Kingdom; Menoufia University, EGYPT

## Abstract

Colon cancer is a significant global health problem, and early detection is critical for improving survival rates. Traditional detection methods, such as colonoscopies, can be invasive and uncomfortable for patients. Machine Learning (ML) algorithms have emerged as a promising approach for non-invasive colon cancer classification using genetic data or patient demographics and medical history. One approach is to use ML to analyse genetic data, or patient demographics and medical history, to predict the likelihood of colon cancer. However, due to the challenges imposed by variable gene expression and the high dimensionality of cancer-related datasets, traditional transductive ML applications have limited accuracy and risk overfitting. In this paper, we propose a new hybrid feature selection model called HMLFSM–Hybrid Machine Learning Feature Selection Model to improve colon cancer gene classification. We developed a multifilter hybrid model including a two-phase feature selection approach, combining Information Gain (IG) and Genetic Algorithms (GA), and minimum Redundancy Maximum Relevance (mRMR) coupling with Particle Swarm Optimization (PSO). We critically tested our model on three colon cancer genetic datasets and found that the new framework outperformed other models with significant accuracy improvements (95%, ~97%, and ~94% accuracies for datasets 1, 2, and 3 respectively). The results show that our approach improves the classification accuracy of colon cancer detection by highlighting important and relevant genes, eliminating irrelevant ones, and revealing the genes that have a direct influence on the classification process. For colon cancer gene analysis, and along with our experiments and literature review, we found that selective input feature extraction prior to feature selection is essential for improving predictive performance.

## 1. Introduction

With the contributions of advanced Machine Learning (ML) technologies, researchers are breaking into healthcare and bio-inspired analysis in datasets of colon cancer cases, which includes biomedical intelligence [1], precision medicine [2], and disease prediction [3]. The wide range of ML models has broadened the scope of the learning process towards hybrid model applications, for instance by integrating an ML model into an optimizer [4] in order to broaden the studied domain, or by implementing physical systems criteria [5] (e.g., medical relevance, context elevations) to remove the low-level hardware barriers of memory and energy overfits.

Cancer research, in particular, is considered one of the most exciting potential areas for ML applications, and intensive efforts have been made to discover possible methods of diagnosis and treatment of cancer [6–8]. However, due to the challenges imposed by variable gene expression, and the availability of open source cancer related datasets which exhibit a high dimensionality, a transductive ML application (e.g., linear regressor [9], Support Vector Machine (SVM) [10], Deep Neural Network (DNN) [11]) with minor feature pre-processing (i.e., feature reduction), if not structured and personalized with the key gene drivers, the transductive learning will not be able to retrieve abnormal cells that express the origin of a tumour. Therefore, the model will risk overfit of data, and the information needed for feature importance could be lost, because the pre-processing structure is not metadata organised with the random growth and the uncontrollable development of cancerous gene features.

The development of microarray technology offers the opportunity for rapid and accurate cancer detection [12,13]. Despite the merits of microarray efficiency in reducing the feature space dimensionality, the promising results of diagnosing and sampling cancer tissues, in particular, are characterized by a small number of spots or probs (small samples of cancer tissue), and external feature transformation techniques [14] and microarray techniques fail to exclude unnecessary features, which result in a low prediction accuracy.

Here, we present a novel two-fold input feature pre-processing approach. The objective is to improve the classification accuracy of colon cancer detection through highlighting important and relevant genes. The purpose behind this model is to select the most related features from the dataset as whole, and eliminate irrelevant ones. This will not only enhance the classification process, but will also show the genes that have direct influence on the classification process.

The first phase of our model consists of feature extraction, which is done by Information Gain (IG) coupled with the popular metaheuristic Genetic Algorithm (GA) used in cancer research to cope with the large number of samples. The second phase is a method for pure gene selection, in which the minimum Redundancy Maximum Relevance (mRMR) filter has been coupled with Particle Swarm Optimizer (PSO) for redundant feature elimination, to narrow down the high complexity of the PSO algorithm. We expand the scope of colon cancer analysis and show the effectiveness of our approach by considering three colon cancer related datasets, then we evaluate our framework model based on the selected features on each of the datasets. We justify the combination of IG-GA and mRMR-PSO according to the computational gaps of each investigated technique, and other criteria based on the feature dimensionality, complexity of colon tissue and the feature subset optimization (see 2.3. Computational gaps).

The rest of the paper is structured as follows: Section 2 consists of the background of our study with the literature sources. Section 3 describes the related previous research work. Section 4 reports the datasets used along with the experiments and some of the characteristics. Section 5 shows the internal design of our framework and highlights the main development steps. While section 6 depicts the process of our conducted experiments, section 7 presents the results of our model with the new proposed feature selection technology. Section 8 evaluates our proposed model using the three datasets and a comparative evaluation with other works. Section 9 draws conclusions and provides insights for future directions.

## 2. Background and literature review

ML is widely used in cancer research and has a high potential in various fields [15]. The use of microarray technologies in cancer research, for extracting features prior to their selection, especially for colon cancer gene classification, remains a hot research topic, due to the nature of cancerous colon tissues and the careful gene selection. The classification of cancer genes helps to improve patient care and supports people through improved quality of life [16–19]. Whereas, the main problem resides in their high redundancy and noise characteristics, which have a negative impact on achieving the high accuracy of cancer classification.

**Fig 1:**
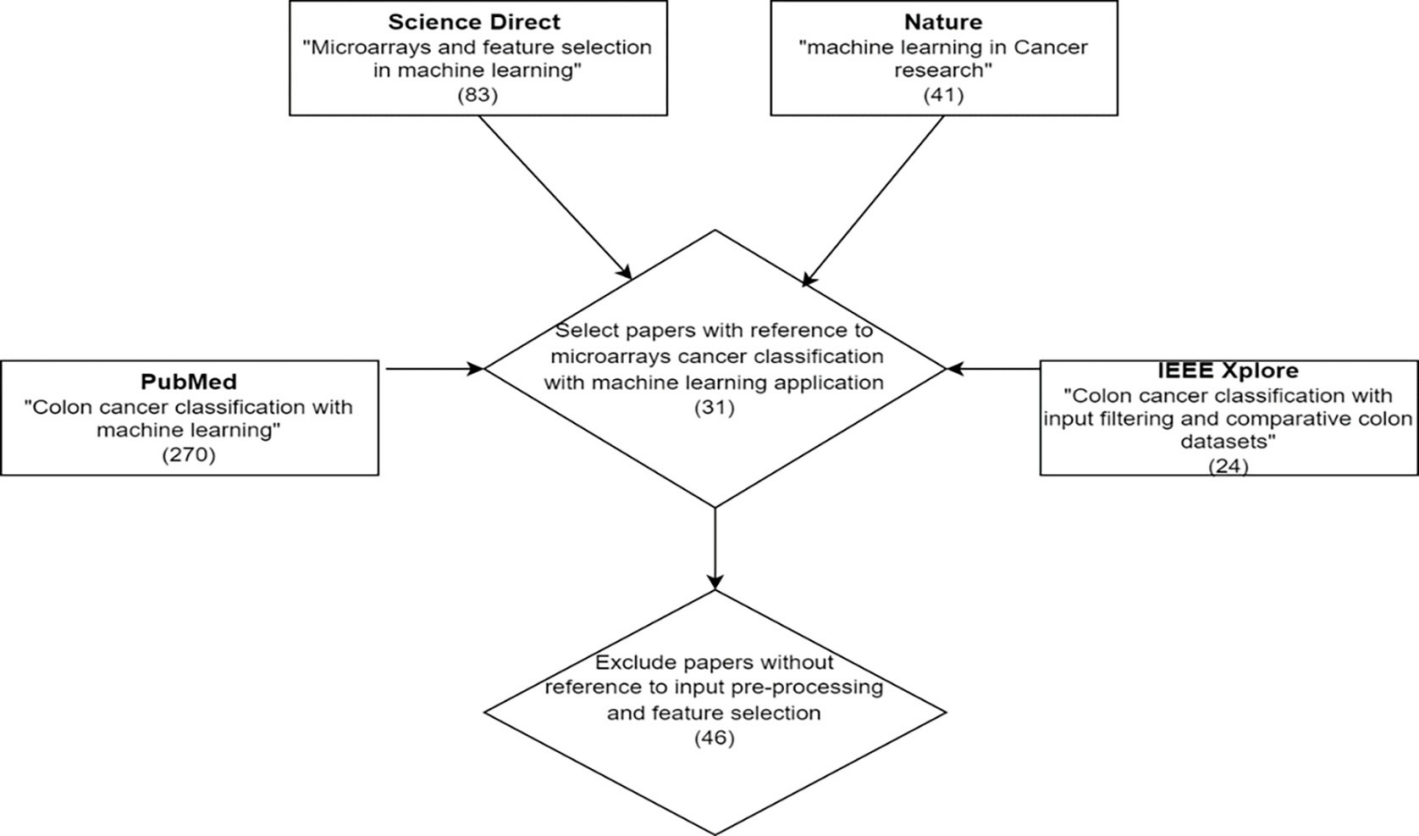
Method of journal papers’ selection.

Our research strategy identifies the main literature resources. Fig 1 illustrates the sources of journal papers consulted throughout the study. A large number of papers about microarrays have been reviewed, and which were identified from academic search engines, such as “Science Direct”, while the journals in “Nature”, “PubMed” and IEEE Xplore were also searched to cover the topic of ML with cancer research and colon research specifically.

### 2.1. IG, GA citations for cancer and colon cancer research

**Fig 2:**
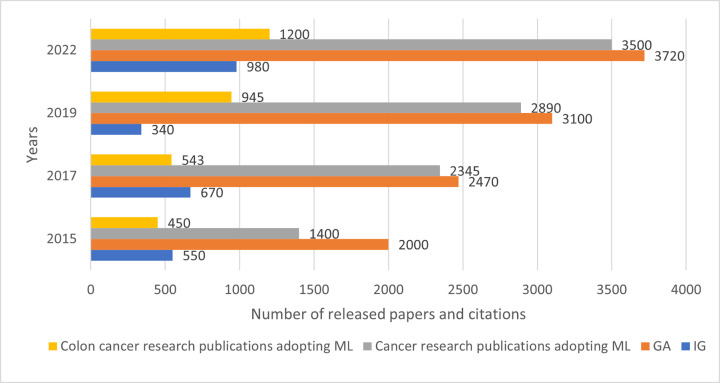
Number of released papers targeting IG and GA for cancer research.

In Fig 2, we plot the number of citations including the proposed feature extraction techniques used in this paper (i.e., IG, GA). We are interested in the publications with IG and GA citations, e.g., papers are published in “Nature” and “Elsevier” through the search engine of ScienceDirect. We used the ASU system (Find Scholarly Works—Arizona State University (elsevier.com)) and libGuide (Citation Count—Using Research Indicators—Library Guides at James Cook University (jcu.edu.au)) for the quantitative analysis.

Fig 2 shows that the number of publications targeting cancer research with ML application has increased between 2015 and 2022, from 1400 to 3500, followed by an increase in publications for colon cancer from 450 to 1250 in the same period. Papers referring to IG have seen an overall increase from 550 to 980, and a steady increase of published papers referring to GA from 2000 to 3720. However, there was a remarkable decrease of published papers referencing IG in 2019 (down from 670 to 340), which was followed by an increase of 980 cited papers in 2022.

### 2.2. mRMR and PSO citations for cancer and colon cancer research

**Fig 3:**
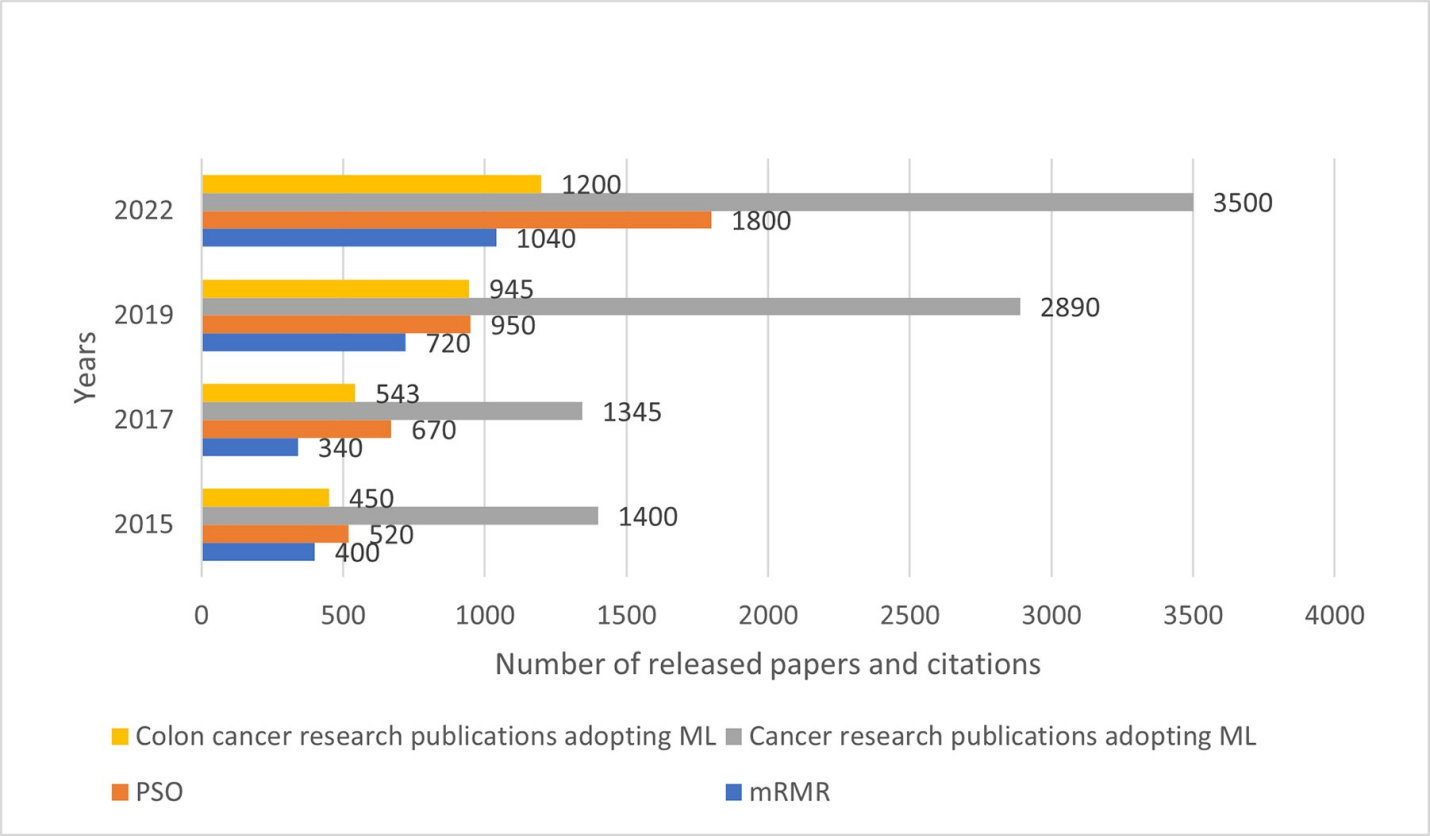
Number of released papers targeting mRMR and PSO for cancer research.

Fig 3 shows the number of citations for the mRMR and PSO techniques, based on the research in cancer and colon cancer, mainly from the journals in “PubMed” and “IEEE Xplore”. Following the logic of the previous illustration (Fig 2), and by considering the trend of released papers, which relate to cancer research and colon cancer in particular, publications that refer to mRMR have seen a slight decline from 400 to 340 between years 2015 and 2017, followed by a sudden increase by the year 2022 (1040 publications). The number of PSO publications follow a rising trend throughout the whole period (500-670-900-1850).

Overall, ML applications for cancer research, and colon cancer research in particular, have been attracting a wide range of research communities over the decades. However, little research work has addressed feature extraction prior to the selection phase. We take this challenge to investigate the accuracy and effectiveness of IG-GA and mRMR-PSO for the early detection of colon cancer. To achieve this objective, we use different ML models with three different colon cancer related datasets.

Touchanti et al. [20] proposed a method based on feature selection for detecting colon cancer. Firstly, they applied the Relief Filter method to rank the genes based on their discriminatory ability and used Recursive Feature Elimination (RFE) to select the best subset of gene expression profiles. Secondly, they used Support Vector Machine (SVM) classification to predict colon cancer, a feed-forward gene selection technique in which two feature selection techniques are used in a sequence. The first technique selects the genes and inputs them into the second feature selection technique. The selected genes are then classified by using linear kernel SVM. Touchanti et al. [20] claimed a gene’s feature reduction resulted from the data augmentation. However, they did not explicitly report the number of genes, neither the number of excluded nor the number of redundant features. Also, in their model design, the two sub-phases for feature pre-processing–data cleaning and normalization–were not discussed.

Mufassirin & Ragel [21] proposed a technique based on a filter-wrapper approach using machine learning methods. They had filtered the data using the class called GainRatioAttibuteEval (in Weka), then the gene subset results were evaluated using a wrapper method [21]. In Mufassirin & Ragel’s study, four colon cancer genes had been selected for training ML models. However, the genes had not been explicitly ranked, and the intermediate phase (i.e., Gene subset) between the proposed filter and wrapper phases was not defined. Moreover, when measuring the model accuracy with or without feature selection, it was not specified which gene attribute among the four selected ones has been excluded or included in the study.

Salem et al. in [22] proposed a method which combines IG and Deep Genetic Algorithm (DGA) approaches. IG was first used to select features, and then DGA was used for feature reduction. Genetic Programming was applied as the classifier for seven different cancers, including colon cancer. Authors in [23] suggested a new hybrid approach to overcome the gene selection issue in cancer classification. They proposed an approach involving use of the mRMR filter and used the binary black hole optimization algorithm (BBHA) to select the discriminatory genes from cancer datasets before applying SVM as a classifier. Despite the explicit gene ranking in [22], the feature dimensionality had been reduced after the necessary genes were selected. The pre-selection step may reduce the important gene expression profile, because the feature reduction will act on specific selected genes, not upon the whole input space, which results in a reduced model accuracy of 85.48%. Therefore, the gene reduction strategy needs to be proceed in the feature selection, in order to preserve the information carried and shared among the important genes. Conversely, the mRMR-BBHA feature selection adopted in [23] had not been framed within a designed model, which made the model’s processing and execution phases difficult to be determined, i.e., either the mRMR technique reduced the genes prior to their optimization, which is done by the BBHA, or the number of features were missed.

Nazari et al. [24] had implemented the lightGBM based Relief Attribute Evaluation and DNN based Relief Attribute Evaluation algorithms to classify colon cancer cells. Furthermore, a study conducted by [25] introduced a hybrid ensemble deep feature selection, high-performance filtering, and an ensemble learning strategy framework. Their model was applied over colon and lung related cancer datasets. The ensemble learning technique proposed by [24,25] showed a good gene expression approximation. However, the pre-classification phase, as presented in [24], including feature extraction, Principal Component Analysis (PCA) and normalization, have either not been tested or justified by the authors. Therefore, the number of features obtained at each step has not been reported. Authors in [25] claimed the use of power spectral density for a pre-processing phase, which was followed by Artificial Bee Colony-Particle Swarm Optimization to improve the classification accuracy. However, there was no evidence for reduced features, nor for the execution trace of the model.

Cahyaningrum et al. [26] introduced the use of the PCA technique to select the most related genes and proposed the use of the Artificial Neural Networks (ANN) and GA hybrid method for cancer classification. The reduction strategy adopted in [26] could be questioned due to the non-quantification of selected or excluded features. Also, the GA algorithm is considered as a post-learning phase, because it happens after the model’s training and testing, so that GA has no impact on the input features. The low predictive accuracy (79.25%) and a slow Area Under the ROC Curve—AUC convergence (82.86%) demonstrated the necessity of enhancing the feature selection process to boost the model’s accuracy.

In addition, authors in [27] introduced a model to overcome the genetic data with high dimensionality issue by applying the Random Subspace (RS) ensemble learning method to five different ML algorithms, namely logistic regression (LR), decision tree (DT), SVM, ANN, and the Bayesian logistic regression FSBRR algorithms. The authors in [27] have tried to mimic the impact of different feature optimizers by defining a redundancy criterion. But again, the model was built on top of uncertainty criteria, where feature redundancy was not accounted for its true value, which abstracts the feature’s importance, i.e., whether or not a feature is carrying necessary cancer gene related information. Shafi et al. [14] introduced a new model which is able to solve the high dimensionality issue and speed up the selection process. They combined the “Mean Decrease Accuracy” (MDA), and the “Mean Decrease Gini” (MDG) as feature selection methods into a well-known classifier known as the Random Forest (RF). The method increases the classification accuracy of the cancer. However, RF as well as K-fold do not provide global search-ability among the important selected genes. The genetic information may be carried on top of genes that do not express a high redundancy, but present a global connectivity that is determined through a fine-tuning global research mechanism such as PSO. Moreover, the authors in [28] proposed a Barnacles Mating Optimizer (BMO) algorithm augmented with Support Vector Machines (SVM) for microarray gene expression profiling to select the most predictive genes for cancer classification. The proposed model is tested on a binary and a multi-class microarray dataset, and the results demonstrate the superiority of the BMO-SVM approach over other meta-heuristic optimization algorithms. Another contribution done by [29] who proposed a hybrid quantum-kernel support vector machine (QKSVM) with Binary Harris hawk optimization (BHHO) called BHHO-PCA-QKSVMfor cancer classification on a quantum simulator. The study aims to improve cancer prediction performance using quantum kernel estimation based on informative genes by BHHO. The colon and breast microarray datasets are used to evaluate the proposed approach’s performance, and the proposed model enhances the overall performance of the two datasets. Another study presented by [30] proposed a hybrid algorithm that combines Manta ray foraging optimization (MRFO) and SVM to select the most predictive and informative genes for cancer classification. The proposed technique was evaluated on binary and multi-class microarray datasets including the colon cancer dataset and compared to other cancer classification algorithms. The results show that the proposed technique achieves high accuracy with the fewest number of informative genes and little effort, making it a promising approach for cancer diagnosis.

Table 1 compares the previous related works based on the data collection technique, the studied tumour, the representative cancerous genes, the feature selection strategy and the respective accuracy of each dataset used, to name few.

**Table 1:** Comparison of the different parameters in previous work.

Author(s)	Data collection technique	Tumour related dataset(s)	Feature selection technique	Representative features	Accuracy (%)
Touchanti et al. [20]	Dataset extraction + sampling from a global dataset.	Colon cancer: 2000 genes with 62 tests: 20 normal and 40 tumoral.	Features filtering using Relief and Recursive Feature Elimination	Not mentioned	99.07
Mufassirin & Ragel [21]	Not mentioned	**Colon**: 2000 genes, **Breast**: 24481 genes, **Lung**:12600, **Leukaemia**: 7129 genes, Ovarian: 15154 genes	Most representative gene subset based on a filter approach + A wrapper subset evaluator.	**Colon cancer dataset**: TGFBR2, CSRP1, MYL9, GUCA2B -Not mentioned for other types of cancer.	95.16 with **Colon cancer,** 100 with **Leukaemia,** 89.69 with **Breast,** 97.04 with **Lung** Cancer and 100 with **Ovarian.**
Salem et al. [22]	DNA microarray dataset as input patterns.	**Leukaemia:** 7129 genes, **Colon:** 2000 genes, **Central nervous system**: 7129 genes, **Lung cancer-Ontario:** 2880 genes, **Lung cancer Michigan**: 7129 genes, **DLBCL**: 7129 genes, **Prostate tumour**: 12600 genes.	Information Gain (IG) for feature selection then Genetic Algorithm (GA) for data reduction.	**Leukaemia cancer**: AML, ALL. **Colon cancer**: GUCA2B, TGFBR2. **Central nervous system**: survivors, failures genes. **Lung-Ontario**: A distant metastasis, disease-free. **Lung-Michigan**: primary adenocarcinomas samples, non-neoplastic samples. **DLBCL**: DLBCL, FL morphology.	97.06 with **Leukaemia.** 85.48 with **Colon tumour.** 86.67 with **Central nervous system.** 74.4 with **Lung cancer-Ontario.** 100 with **Lung cancer-Michigan.** 94.80 with **DLBCL.** 100 with **Prostate cancer.**
Pashaei et al. [23]	GSE70768-9, GSE25136, BPSO (4–2)/BBHA/SPLSD microarrays for input genes sampling.	**Breast cancer:** 24481 genes **Central nervous system:** 7129	Binary Black Hole Algorithm (BBHA) + Particle Swarm Optimisation (PSO) (4–2)	**Breast cancer: AL080059, AF055033,** **Contig412_RC,** **NM_018964** **Central nervous system: J02611_at, HG2994-HT4850_s_at,** **S71824_at,** **M13194_at**	92.16 with **Breast cancer** and 99.33 with **Central nervous system.**
Nazari et al. [24]	Microarray from GEO, NCBI databases.	**Colon cancer** dataset.	RNA extraction from the cancer tissue: PCA for dimensionality reduction, data matrix normalization and balancing.	Not mentioned.	100 with **Colon cancer** with DNN application.
Talukdar et al. [25]	Histopathological image datasets (LC25000) have been taken for lung and colon and combine both (lung and colon) cancer datasets.	**Lung cancer:** 4200 images. **Colon cancer:** 2800 images.	Use transfer learning coupled with a deep convolutional approach for feature extraction,	**Lung Cancer:** lung_aca, lung_n, lung_scc **Colon cancer:** colon_aca. Colon_n.	99.05 with **Lung cancer,** 100 with **colon cancer** and 99.30 with **Lung + Colon cancer.**
Cahyaning-rum et al. [26]	Microarray sampling of cancer tissue.	**Colon tumor:** 2000 genes **Prostate tumor:** 12600 genes **Lung cancer:** 12533 genes	PCA dimensionality reduction, eigenvalues ordering, and then Min-max normalization.	Positive vs negative, normal vs tumor feature sampling for **Colon** and **Prostate tumor** datasets respectively. Mesothelioma vs ADCA for **Lung cancer.**	Improved accuracy reported with 15 hidden neurons: 83.33 with **Colon cancer,** 76.47 with **Prostate cancer** and 89.93 with **Lung cancer.**
Zhang & Cao [27]	Not mentioned.	**Colon tumour:** 2000 genes **Nervous system:** 7129 genes **DLBCL-Stanford:** 4026 genes, **P53 Mutants: 5409** genes, **Arcene:** 10000 genes, **BRCA:** 21548 genes **GBM:** 18348 genes **TSP:** 319 genes	filter feature selection algorithm based on redundant removal (FSBRR).	Not mentioned.	92.01 with **Colon cancer,** 80.17 with **Nervous system,** 83.66 with **DLBCL-Stanford,** 94.31 **with p5 Mutants,** 855.67 with **Arcene,** 86.26 with **BRCA,** 82.03 with **GBM** and 77.69 with **TSP.**

### 2.3. Computational gaps

When it comes to feature selection, factors like feature dimensionality, biased features, optimizers, etc., which represent a serious bottleneck towards an explainable and transparent feature selection. By the following, we want to emphasize the significance of the targeted feature selection problem in this paper, we surround and justify the proposed techniques, and provide a brief of scientific challenges related to the colon cancer research in particular.

Although Information Gain (IG) has been proven for an accurate feature selection, it fails to cope with higher distinctive features. Because filter models, including IG in particular, are known for a perfect separation of feature selection and the classification process, a natural bias always dominates the training data, since a broad tree will be constructed to structure the highest possible values of an attribute. Therefore, the resulting classifier will perform poorly in predicting unknown instances, e.g., colon tumour detection in [22].

The Genetic Algorithm (GA) does not require highly distinctive features to perform well. However, this makes GA hard to be implemented and adjusted to the study case, e.g., colon tumour analysis [31–33]. Slow convergence time, initialization problems and local optima optimizations are the common disadvantages of GA. Therefore, we have decided in this research to combine GA with IG to cope with higher dimensionality, as well as the high distinctive colon tissue features by reducing the randomness of genetic feature subsets generated from the GA, and improve the fitness of each binary set. This combination is also justified by the trend of published papers (see Fig 2) targeting GA and IG.

In order to broad the search ability and selection done by GA, i.e., a feature subsets analysis towards a global optimization, we use Particle Swarm Optimization (PSO). However, due to the high dimensionality of colon cancer features, and because the generated features-subsets which are imbalanced and uncorrelated to the colon cancer target variable. For that reason, we implement the (minimum-Redundancy Maximum Relevance (mRMR) feature selector, in which a relevance score is applied to calculate the correlation between the target variable and other features, and a redundancy score to define a feature ordering and eliminate the redundant, non-informative subset of features. However, we join mRMR and PSO because the constituent genes of colon tissue have variant representative features (see Table 1), and the segmented cell tissue provides multiple variants (e.g., TGFBR2, CSRP1, MYL9, etc.) which fail to be locally scored with a high relevance and optimized. Thus, we use PSO for a global variant subset optimization. As illustrated by Fig 3, there was a high correlation in the recent years between released papers targeting colon cancer research and the ones using PSO, mRMR for the feature selection purpose.

## 3. Dataset reporting

Biological Samples of colon dataset are normally collected from patients and then processed by microarray technology to be ready for analysis. Currently, it is difficult to obtain the data on the human genome from a central database [27,34–36]. However, there are many publicly available genetic expression datasets that are commonly used in cancer selection and classification experiments. In this paper, we collected the data from three publicly available colon cancer datasets.

The first dataset collected from Alon et al. [37] has been used in several studies of colon cancer [27,31,32,33,35,36,38–45]. The dataset is composed of 2000 genes (attributes) and 62 samples (cases) taken from colon cancer patients. This dataset was original composed of 6000 gene expressions. However, 4000 genes were eliminated based on the confidence in the expression levels measured. There are 40 samples categorized as abnormal cells (tumour biopsies), and 22 are normal cells (benign biopsies).

The second dataset collected from Notterman [46] has also been used in recent studies [38,44,45]. This dataset is composed of 7457 genes (attributes), and 36 samples (cases) taken from colon cancer patients. There are 18 samples categorized as abnormal cells (tumour biopsies), and 18 are normal cells (benign biopsies).

The third and last dataset collected from GEO at NCBI (Gene Expression Omnibus at the National Centre for Biotechnology Information) [47] has also been used in recent studies [40]. This dataset is composed of 22278 genes (attributes), and 111 samples (cases) taken from colon cancer patients. There are 56 samples categorized as abnormal cells (tumour biopsies), and 55 are normal cells (benign biopsies). This dataset was collected and saved using MS Excel, and then transformed using Python language. The details of the datasets are described in Table 2.

**Table 2:** Gene expression datasets used in the investigations.

Type of Dataset	No. of Genes across the samples	Classification Type	No. of Samples	
Alon et al. (KentRidge) [37]	2000	Tumour	62	40
		Normal		22
Notterman [46]	7457	Tumour	36	18
		Normal		18
GEO at NCBI [47]	22278	Tumour	111	56
		Normal		55

**Fig 4:**
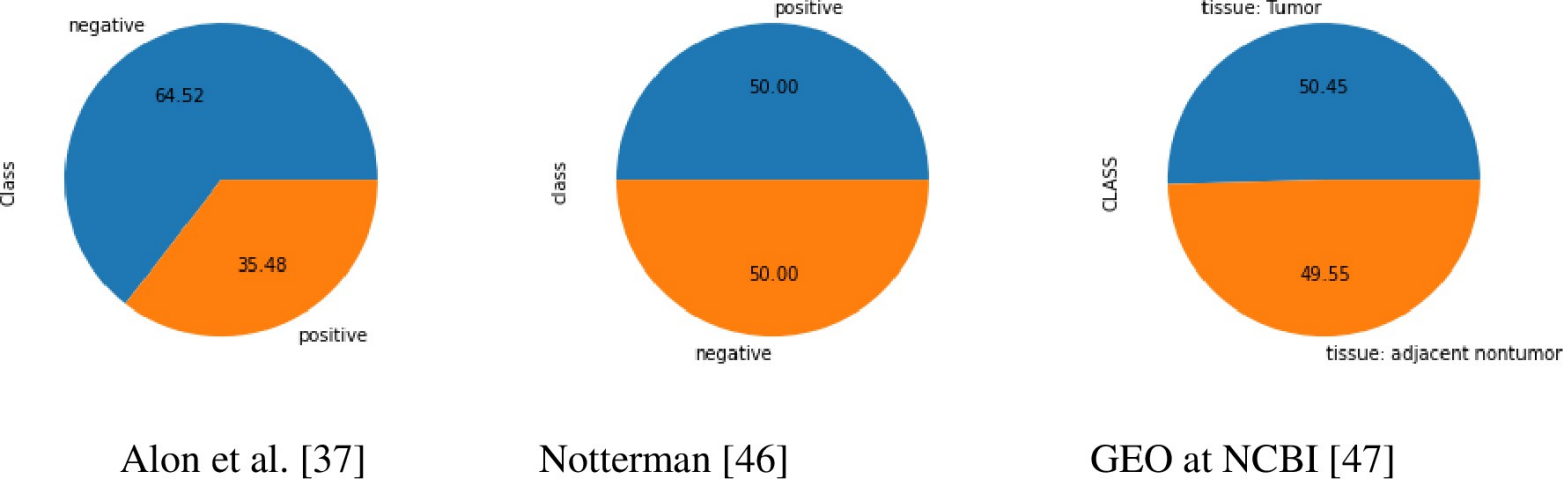
Class label distribution for the colon cancer datasets.

In Fig 4 the number of samples from the first dataset is 62 distributed as 40 tumour tissues and 22 normal tissues. This is an imbalanced classification distribution that may affect the machine learning training model by a little skew or bias. To avoid this issue, different evaluation techniques (such as recall, precision, and F1-Score) will be used in order to make sure there will be no overfitting or underfitting for this dataset. The other two datasets are balanced and ready to be pre-processed. However, in the second dataset (Notterman) [46] class labels are ordered as the first 18 positive labels comes before the second 18 negative labels. In order to avoid this issue and to make sure that there is no bias in the classification, we shuffled the sample records.

PCA is a statistical technique that uses symmetrical transformation. The observations of a potential correlation variable are converted to the values of a linearly uncorrelated variable, called the main component (or sometimes called the principal modes of variation). PCA applies to data tables that represent observations of several dependent variables that are generally intercorrelated. The aim is to extract relevant data from the data table and represent the information in the form of a new orthogonal variable set.

**Fig 5:**
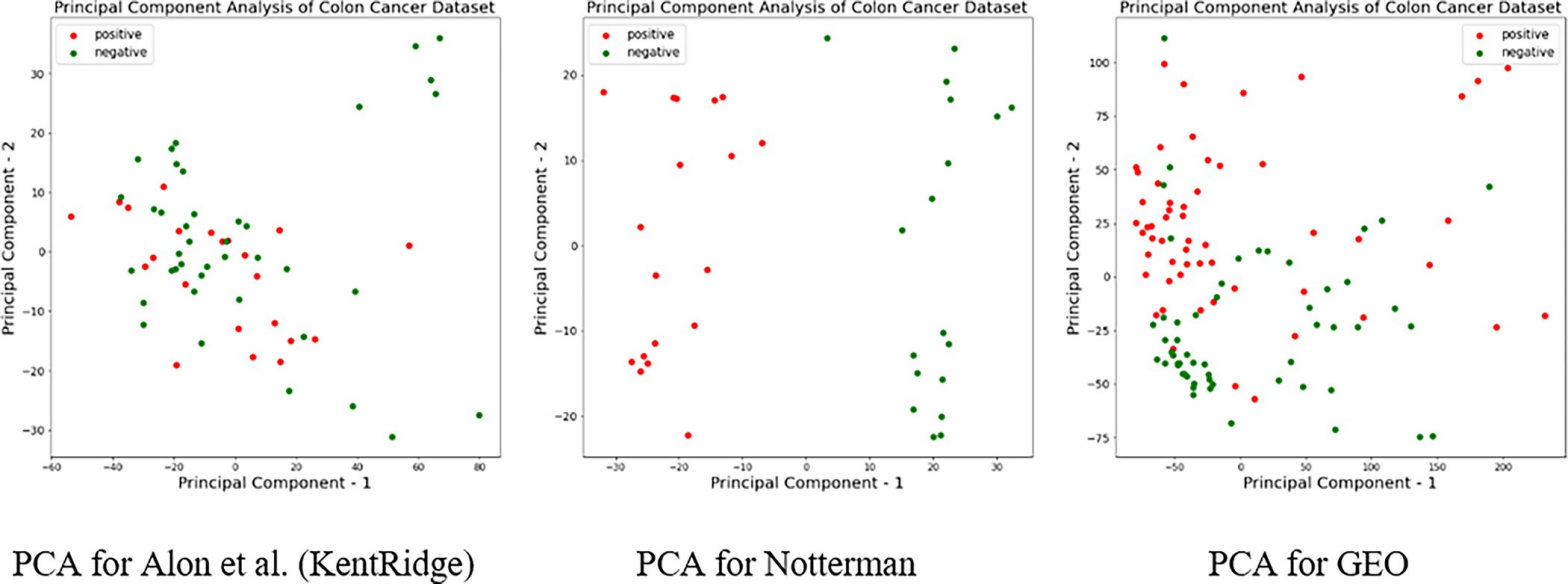
PCA for exploring the data structure of the datasets.

Fig 5 visualizes and identifies each colon tissue sample with different colours for an easy observation. We can see how this method separates different types of tissue samples from each set and how the use of PCA allows us to identify the data structure.

Fig 5 illustrates the sample data structure of dataset 1 (Alon et al. (KentRidge)) [37] from different classes, which are not distinct, while the samples data structure of dataset 2 (Notterman) [46] from the different classes are more distinctive and easily separable. However, the samples in the data structure of the third dataset (GEO at NCBI) [47] from different classes are less distinctive.

## 4. Methodology

### 4.1 Feature selection algorithms

The main contribution of this research is the development of two phases model—HMLFSM. The first one is composed of a feature selection algorithm prior to classification. The second phase is to apply hybrid feature selection algorithms. The objective is to improve the classification accuracy of colon cancer detection and to highlight important and relevant genes. The purpose behind this framework model is to select the most related features from the dataset as whole, and eliminate non-relevant ones. This will not only enhance the classification process, but also will show those genes that have direct influence on the classification process. In this study we propose the use of the following feature selection algorithms because they showed significant impact when applied to genetic data specially for the colon cancer datasets as stated in section 2 earlier.

#### 1. Information Gain (IG)

Information Gain is a popular feature selection method that assesses each feature’s relevance to the target variable. There are numerous factors that can influence colon cancer, including age, gender, and lifestyle. We can identify the most relevant features that contribute to the development and progression of colon cancer using Information Gain. IG calculates the reduction in entropy (i.e., the amount of uncertainty or randomness in the dataset) that results from using a particular feature to split the data into smaller subsets. IG can be formulated as follows:

IG=Entropy(parent)‐[WeightedaverageofEntropy(child)]

For example, if we want to predict whether a patient has colon cancer based on their age, gender, and lifestyle habits, Information Gain would help us identify which of these features is the most informative for predicting the presence of colon cancer. It does this by comparing the amount of uncertainty in the parent dataset (i.e., the entire dataset before any splits are made) to the amount of uncertainty in the child datasets resulting from splitting the parent dataset based on a particular feature. The feature that results in the greatest reduction in uncertainty (i.e., the highest Information Gain) is considered the most relevant for predicting the target variable.

#### 2. Genetic algorithms (GA)

Are a powerful optimization technique inspired by the natural selection process. They are especially useful for feature selection in high-dimensional datasets, where the number of features is larger than the number of samples. Using genetic algorithms, we can efficiently search for the optimal subset of features that maximizes colon cancer classification accuracy. The first step in applying GA to colon cancer classification is to represent the genetic data as a set of binary strings. Each binary string represents a set of genetic features that can be used to predict the presence or absence of colon cancer. The next step is to create an initial population of potential solutions by randomly generating a set of binary strings. Each binary string represents a possible combination of genetic features that could be relevant to colon cancer classification. The population is then evaluated based on how well each individual solution (i.e., binary string) classifies the genetic data. This evaluation is done using a fitness function that calculates the accuracy of each solution. The most accurate individuals (i.e., binary strings) are then selected as parents for the next generation. These individuals are subjected to genetic operators such as crossover and mutation, which introduce variation into the population and create new binary strings. The resulting offspring form a new generation of solutions that are evaluated and selected for the next round of reproduction. This process continues until a satisfactory solution is found or a stopping criterion is met. The final solution is the binary string that accurately classifies the genetic data with the highest fitness score.

#### 3. Minimum Redundancy Maximum Relevance (mRMR) and Particle Swarm Optimization (PSO)

Are two feature selection methods that have been shown to perform well. mRMR selects features with high relevance to the target variable while minimizing feature redundancy. PSO is a global optimization algorithm that can search the feature space efficiently to find the best subset of features. We can improve feature selection performance and classification accuracy in colon cancer by combining mRMR and PSO.

The mRMR algorithm computes two scores for each feature in the dataset: a relevance score, which measures how much the feature is correlated with the target variable (i.e., colon cancer), and a redundancy score, which measures how much the feature is correlated with other features in the dataset. The algorithm then selects the features with the highest relevance and lowest redundancy scores to form a subset of informative and non-redundant features. The PSO algorithm is then used to optimize the subset of selected features for classification accuracy. PSO is a population-based optimization method that searches for the optimal solution by iteratively updating a set of candidate solutions, known as particles, based on their fitness scores. In the context of colon cancer classification, the fitness score measures the accuracy of a particular subset of selected features in classifying the genetic data. The PSO algorithm seeks to find the subset of selected features that maximizes the classification accuracy. The combination of mRMR and PSO involves iteratively selecting a subset of informative and non-redundant features using the mRMR algorithm and then optimizing the subset using the PSO algorithm. The process continues until a satisfactory subset of features is found or a stopping criterion is met.

In summary, the implementation of these algorithms provides a robust and effective approach to feature selection in colon cancer, assisting in the identification of the most relevant features for accurate disease diagnosis and treatment.

### 4.2 System design

**Fig 6:**
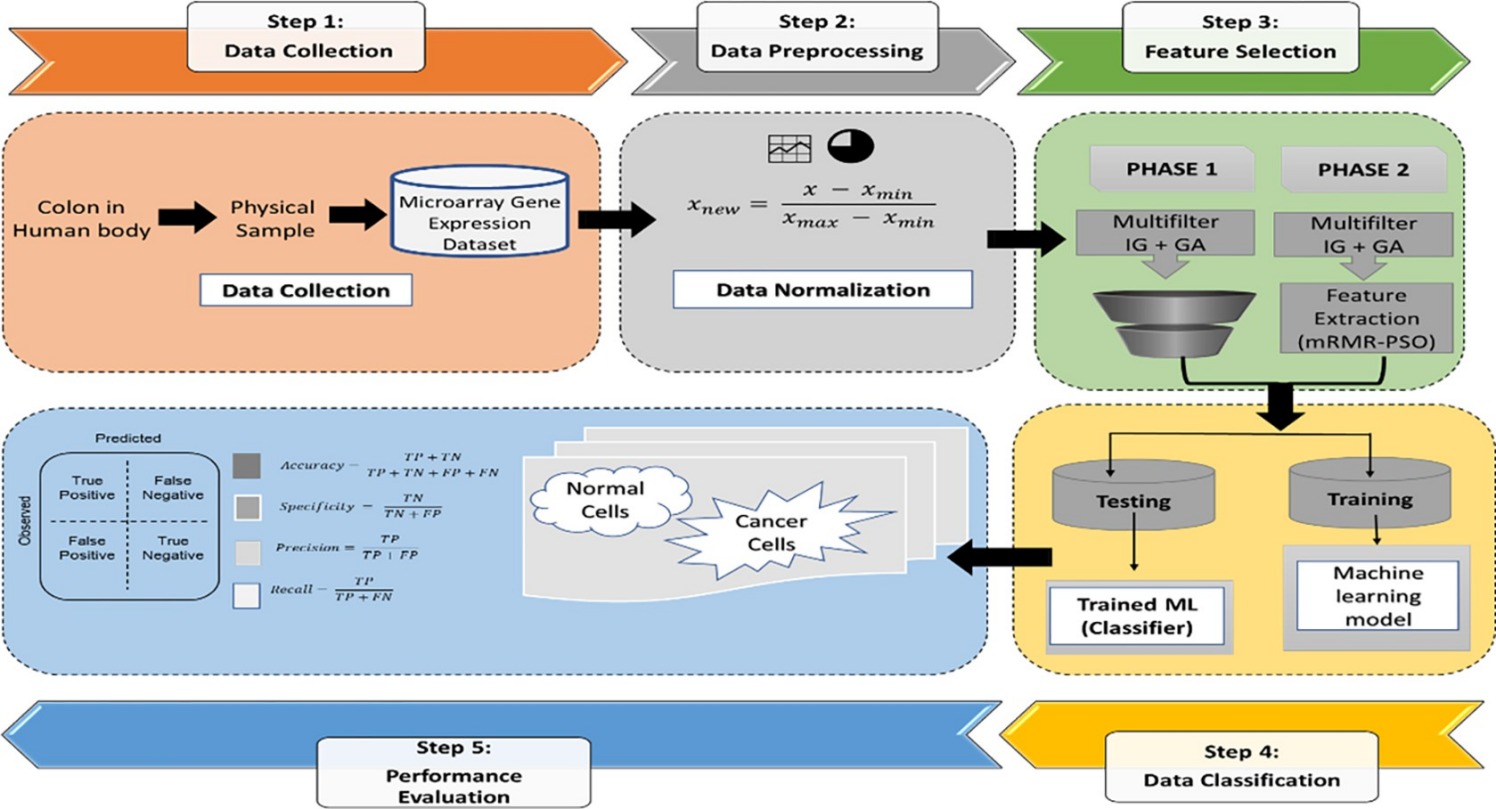
The proposed framework model–HMLFSM.

As presented in Fig 6, the process starts with the data collection as microarray gene expression. Then the framework model is divided into two phases as follows:

1. In the first phase, a multifilter hybrid set of IG and GA feature extraction algorithms is applied. The output of this phase is trained and tested using ML algorithms, then the results are validated and recorded.
2. In the second phase, the mRMR algorithm is applied in addition to the multifilter hybrid feature selection algorithms (IG + GA) to extract top features from the data set, then the deep learning classifier is executed, and the results are validated and recorded.

However, a comparative study with and without feature selection has been carried out for comparison, with same method tested using different ML algorithms. The proposed framework model HMLFSM–Hybrid Machine Learning Feature Selection Model in Fig 6 consists of 5 steps:

- **Step 1:** Collecting the data from different resources (3 datasets) using microarray technology
- **Step 2:** Dataset pre-processing
- **Step 3:** Feature selection
- **Step 4:** Training and testing machine learning models
- **Step 5:** Performance evaluation and recording the results.

## 5. Experiments

To prepare the experiments, the following prerequisites–data preparation (pre-processing, in addition to dataset reporting in section 4), environment set-up, and the execution process arriving to the model design–are defined as follows.

### 5.1 Dataset preparation

The pre-processing of datasets (see section 4) is considered as an important step to work with gene expression data. The colon cancer datasets are normalized to balance the intensity of hybridization of each point of the data matrix, and then carried out so that each gene expression has a mean equal to zero and a variance equal to 1 [7]. We normalise the first two datasets (Alon et al. (KentRidge) [37] and (Notterman) [46]), while the third dataset (GEO at NCBI) [47], is already normalised in the source. However, the second dataset [39] is initially pre-processed by eliminating any duplicated genes to keep only the unique ones, and then each array is standardised into zero mean and unit variance [17]. 860 duplicates were found and removed.

A problem that must be overcome in a genetic study is that the set of analysis data is small relative to the entire genetic population. In addition, global genetic datasets are characterized by “noise” and redundant information [14]. The use of feature filtering technology is considered as a way of solving this situation by preparing the raw data in an appropriate analytical form.

Before conducting the classification process, the data must be split into training and testing sets. It is important that a testing set be independent to enhance and improve the validity of the classification accuracy and to validate the model [17]. In our experiments, we divided the first [37] and the third [47] datasets into 70% of the samples for training and the remaining (30%) for testing. This is because there were more samples in these datasets. On the other hand, we applied 10-fold cross validation for the second dataset [46].

### 5.2 Environment set-up

The WEKA (3.8.5) machine learning workbench (https://ai.waikato.ac.nz/weka/) was used in this research, since Weka resources provide extensive library for machine learning algorithms as well as many techniques for data validation [38]. In addition, the Python programming language was employed for data pre-processing and visualization. Various Python libraries have been employed, including Scikit-learn and Matplotlib. Matplotlib was utilized to visualize the data, while the Scikit-learn library was utilized for pre-processing and implementing machine learning algorithms. The computing environment used a PC with the Windows 10 operating system, an Intel(R) Core (TM) i7 CPU and 16 GB of installed RAM.

### 5.3. Experimental design

The experimental process is composed of two phases using several ML algorithms for feature selection and classification (Fig 6). The process is described below.

- **First phase:** a hybridisation between the Information Gain (IG) and Genetic Algorithm (GA) models are applied after the data being pre-processed. The IG algorithm is implemented to generate discriminatory scores for each gene and eliminating all zero-point genes from the data set. Then, using GA, many information-rich genes are selected to optimize datasets using informative and correlated genes. Finally, a list of the most common classification algorithms (Decision Trees, Random Forest, Support Vector Machine, K-Nearest Neighbours, Naïve Bayes, and the Deep Neural Networks) are applied on the selected data.
- **Second Phase:** The redundancy level is further reduced by minimum Redundancy Maximum Relevance (mRMR) algorithm in order to maximize the efficiency of gene selection processes and keep only more relevant genes by searching for the optimised ones. The goal is to minimize the number of analyses and reduce the amount of data “noise”.

## 6. Results and discussion

### 6.1. Number of selected features

Since the proposed framework model consists of two phases, Table 3 shows the number of the most relevant features selected at each phase. By applying phase 1 the numbers of relevant features selected were 68, 459, and 2206 for datasets 1, 2, and 3 respectively, thus reducing the dataset sizes by around 96.6%, 93.8%, and 90.1% respectively. However, when phase 2 is applied the numbers of genes are reduced to 22, 35, and 68 features, which represents almost 99%, 99.5%, and 99.7% of the original datasets, leaving only those relevant genes.

**Table 3:** The number of relevant genes (features) selected by the proposed model on each phase.

Datasets	Complete Dataset	Number of Selected Features	
		Phase 1: (Hybrid IG + GA)	Phase 2: Phase 1 + (mRMR-PSO)
Alon et al. (KentRidge) [37]	2000	68	22
Notterman [46]	7457	459	35
GEO at NCBI [47]	22278	2206	68

### 6.2 Model performance with (IG + GA) and (mRMR + PSO)

In this section, the classification accuracy and validation results for the proposed model are presented and discussed.

- **Dataset 1:**

**Fig 7:**
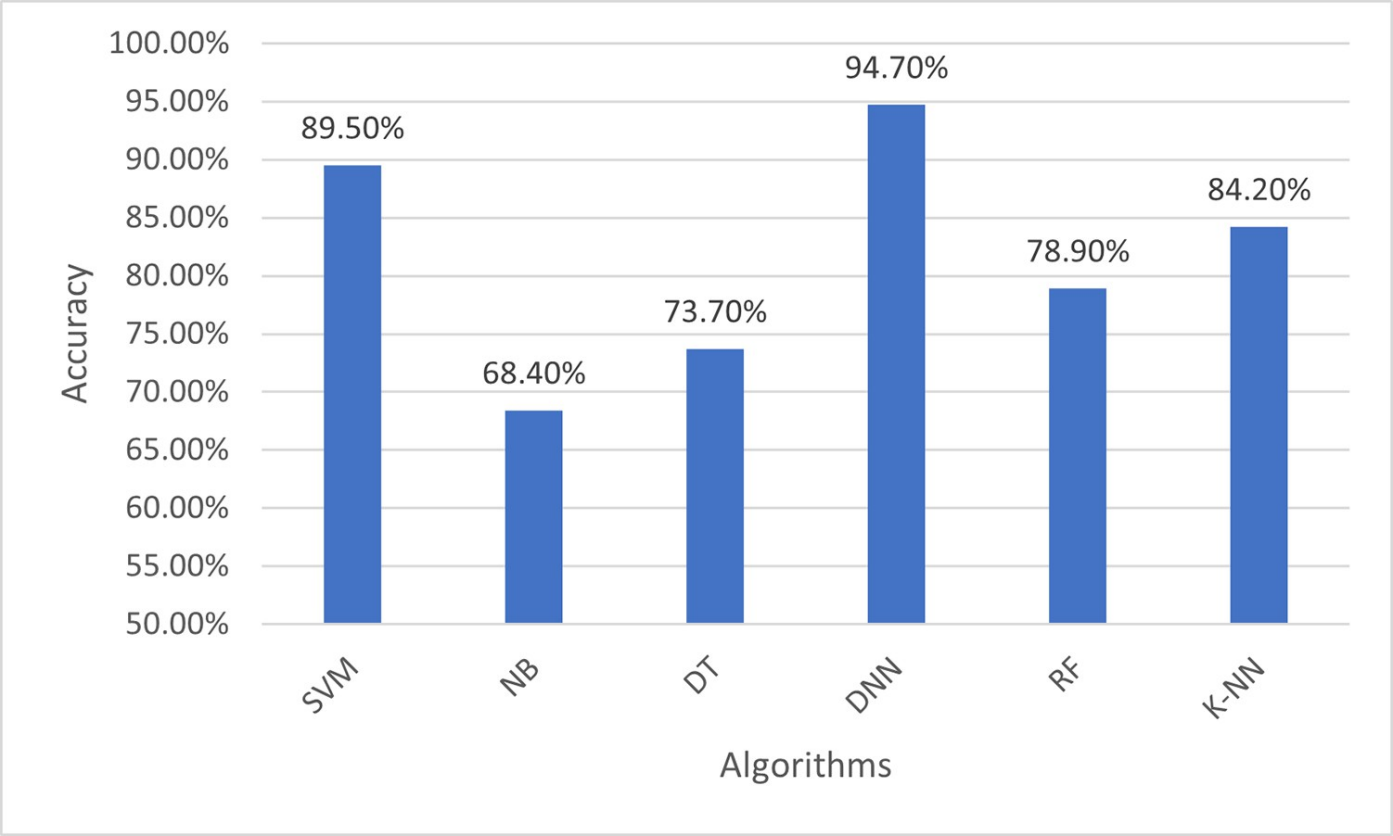
Classification accuracy for the ML algorithms applied to Dataset 1.

**Fig 8:**
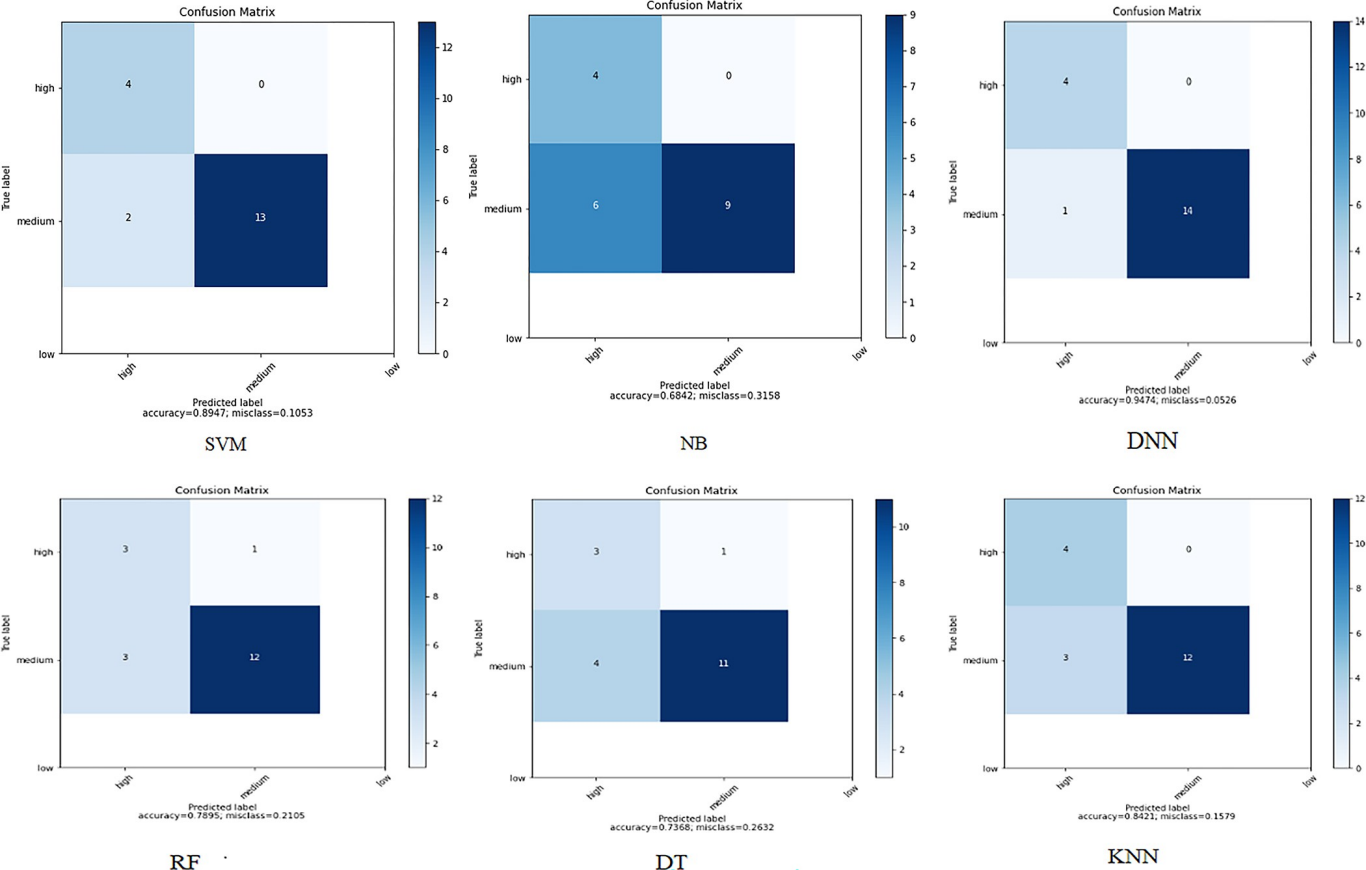
ML confusion matrix for the ML algorithms applied to Dataset 1.

**Fig 9:**
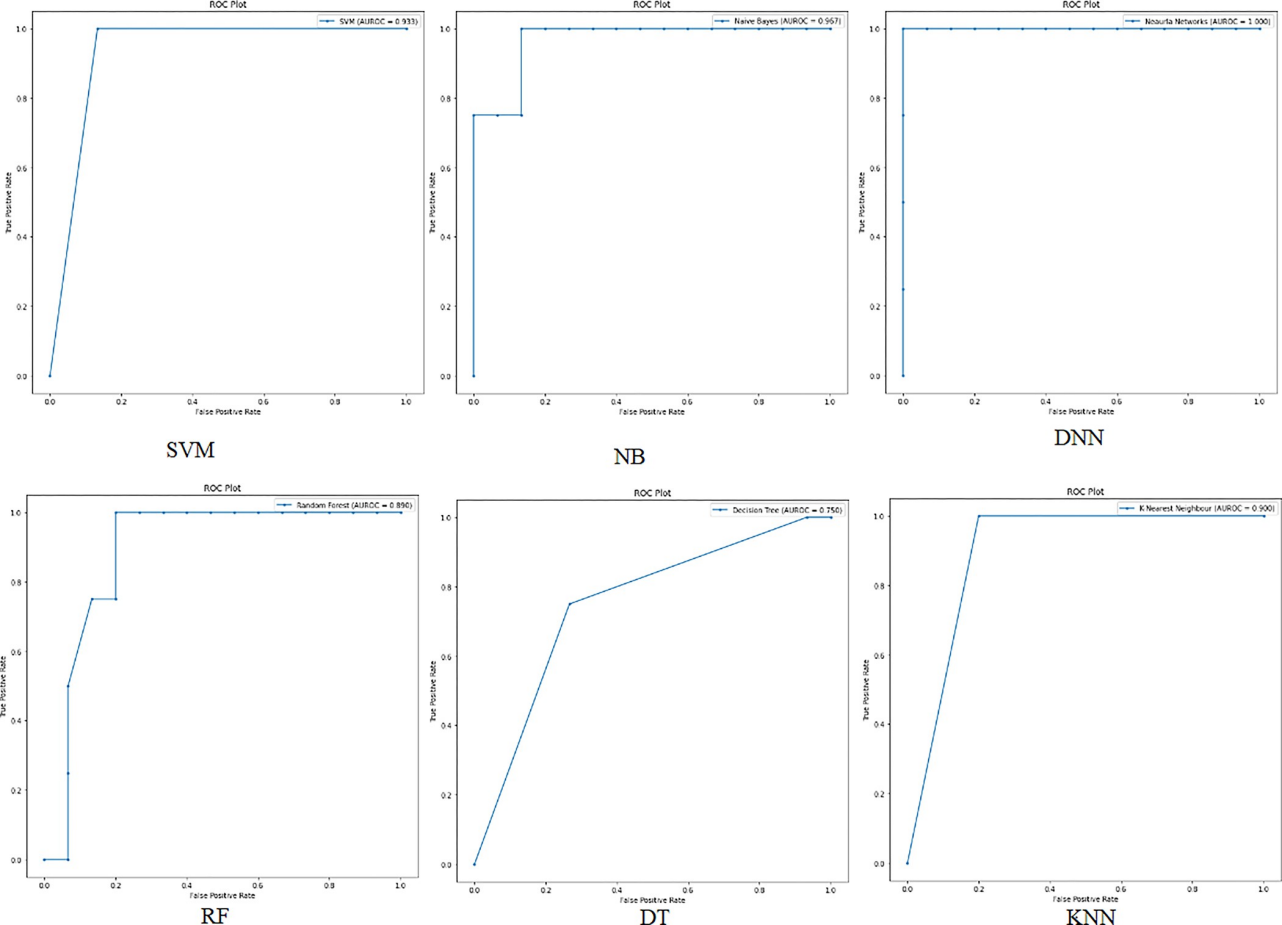
ROC area for the ML algorithms applied to Dataset 1.

When applying the proposed model to the first colon cancer dataset (Alon et al. [37]), DNN has a promising accuracy of almost (95%) with high Receiver Operating Characteristic (ROC) area which plots the true positive rate (sensitivity) against the false positive rate. Figs 7 and 8 represent the prediction accuracies and confusion matrices, respectively, for the ML algorithms applied to this dataset. Fig 9 presents the Area Under the ROC Curve (AUC) to evaluate the classification performance of the ML algorithms applied to the same dataset. It measures the probability that the model will correctly identify a positive example as having a higher predicted probability of being positively compared to a randomly selected negative example. The AUC illustrates how DNN has a high area of the curve while classifying the positive cases almost 1.

- **Dataset 2:**

**Fig 10:**
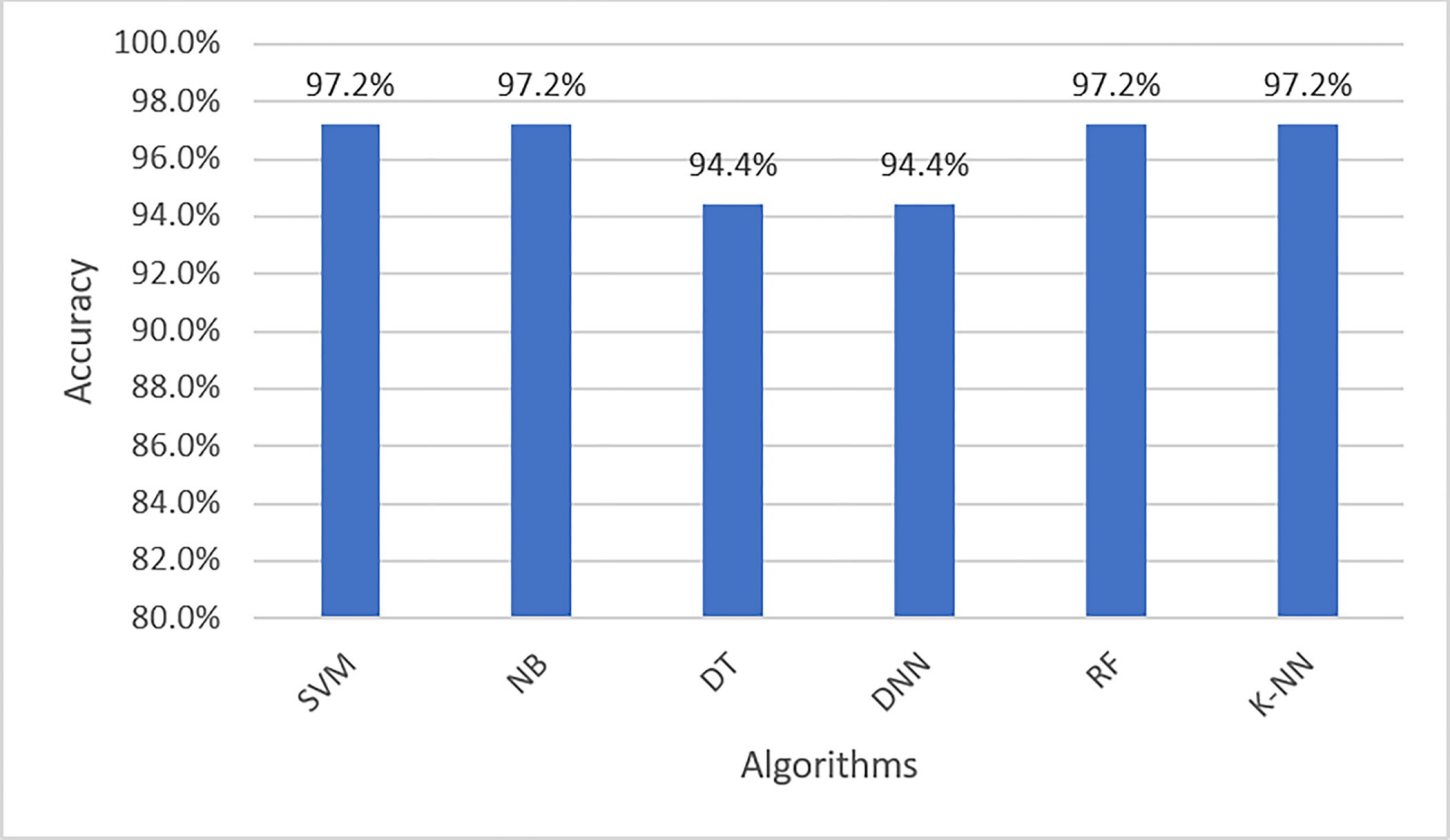
Classification accuracy for the ML algorithms applied to Dataset 2.

**Fig 11:**
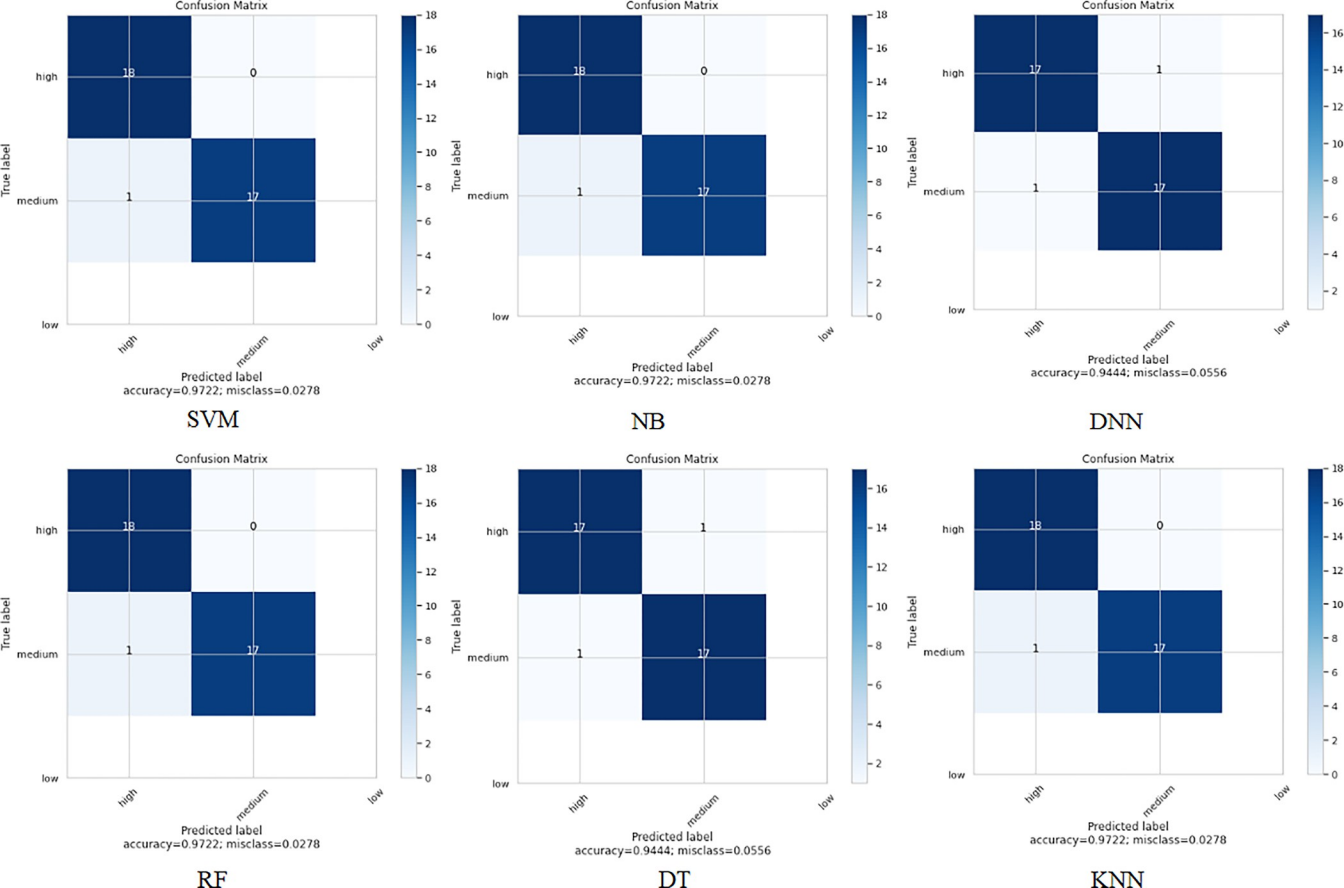
Confusion matrix for the ML algorithms applied to Dataset 2.

**Fig 12:**
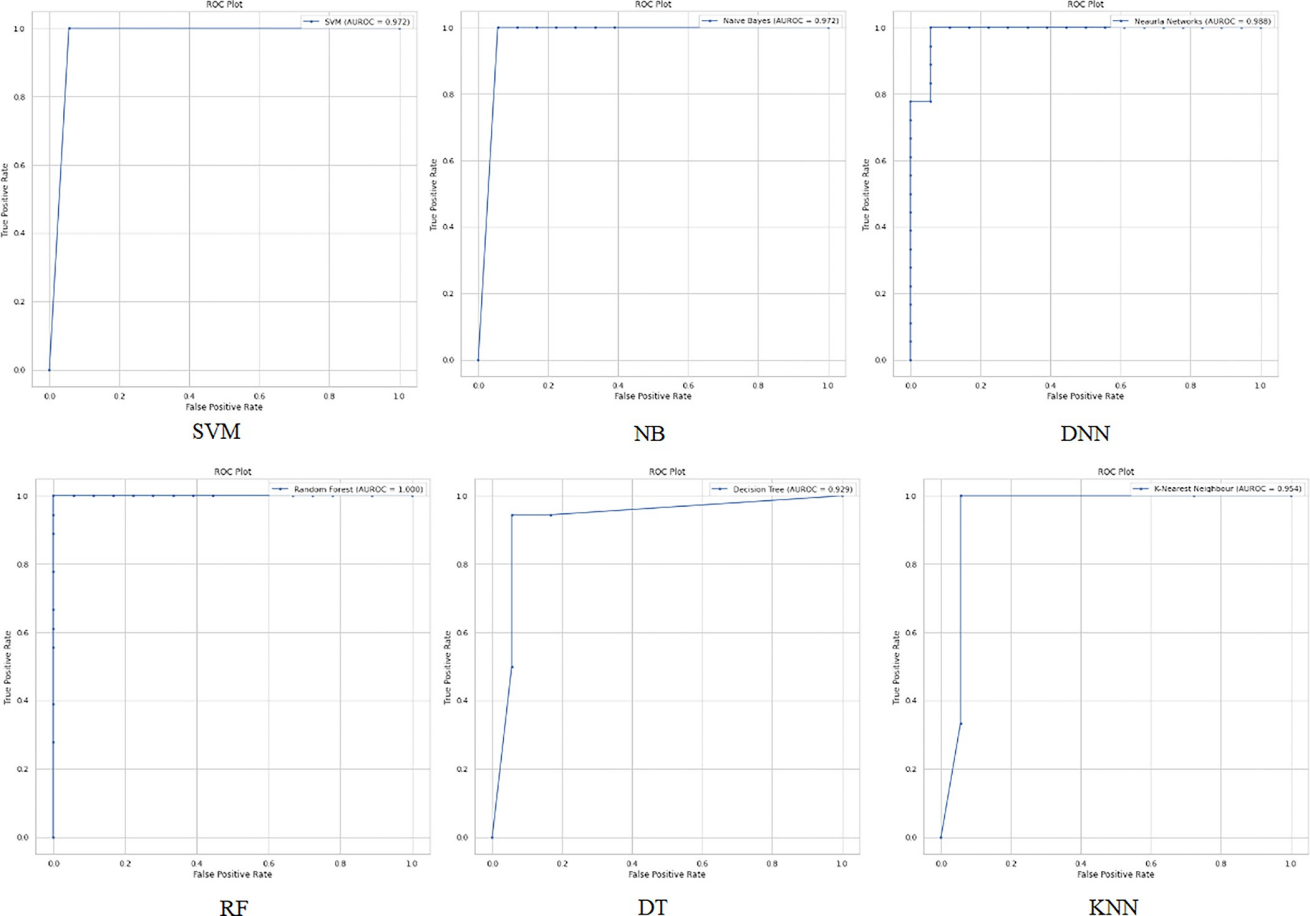
ROC area for the ML algorithms applied to Dataset 2.

When applying the proposed model to the second colon cancer dataset (Notterman) [46], the reduced number of samples compared to the other two datasets prevented the accuracy from being improved for the DNN and DT algorithms. We observe that SVM, NB, RF, and KNN have the highest classification accuracy (97%). However, DNN still has promising accuracy results (94%). Figs 10 and 11 depict the accuracy and confusion matrices, respectively, for the ML algorithms applied to this dataset, and Fig 12 illustrates the ROC measure for the classification of the machine learning algorithms applied to the same dataset. It is clearly noticed that RF and DNN have a high area of the curve classifying the positive cases.

- **Dataset 3:**

**Fig 13:**
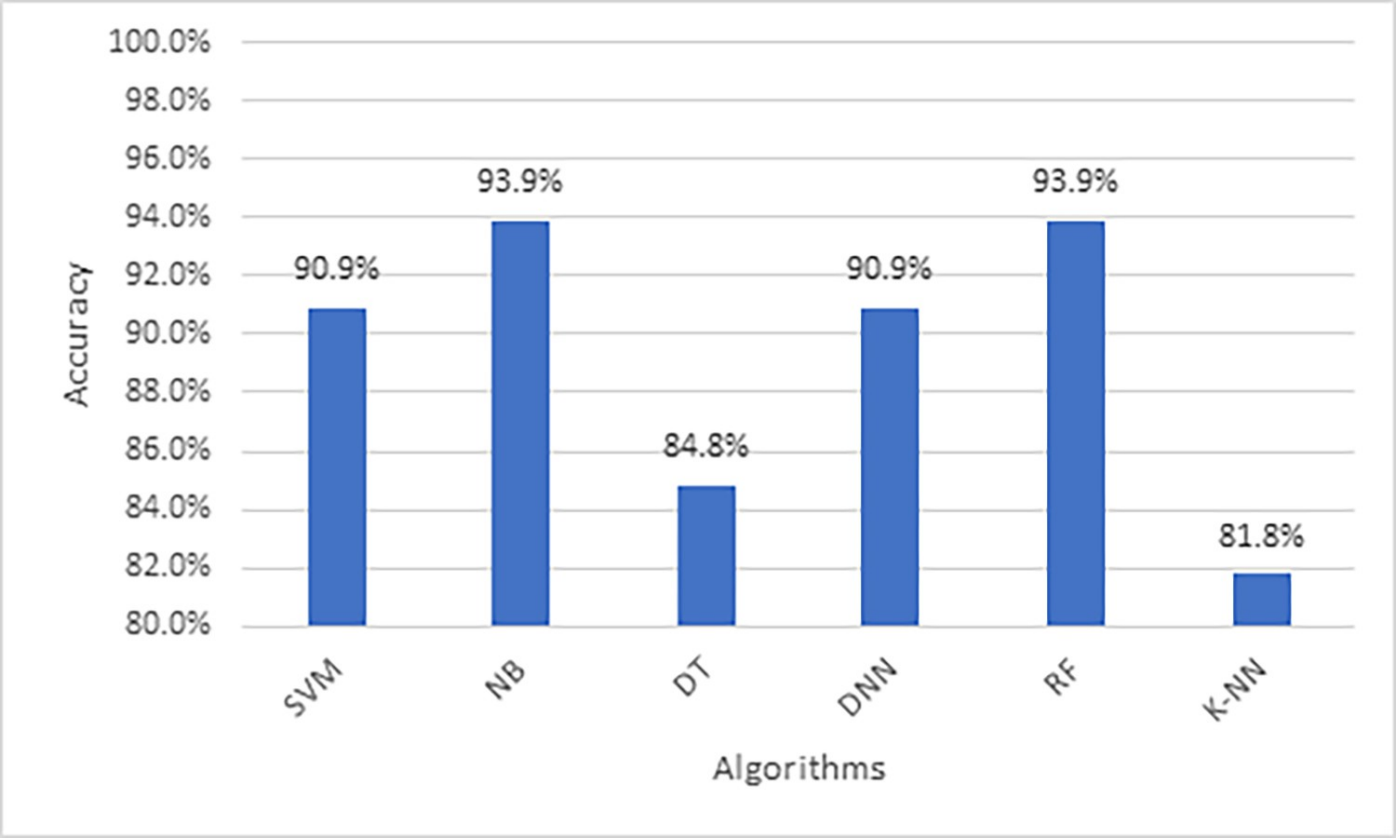
Classification accuracy for the ML algorithms applied to Dataset 3.

**Fig 14:**
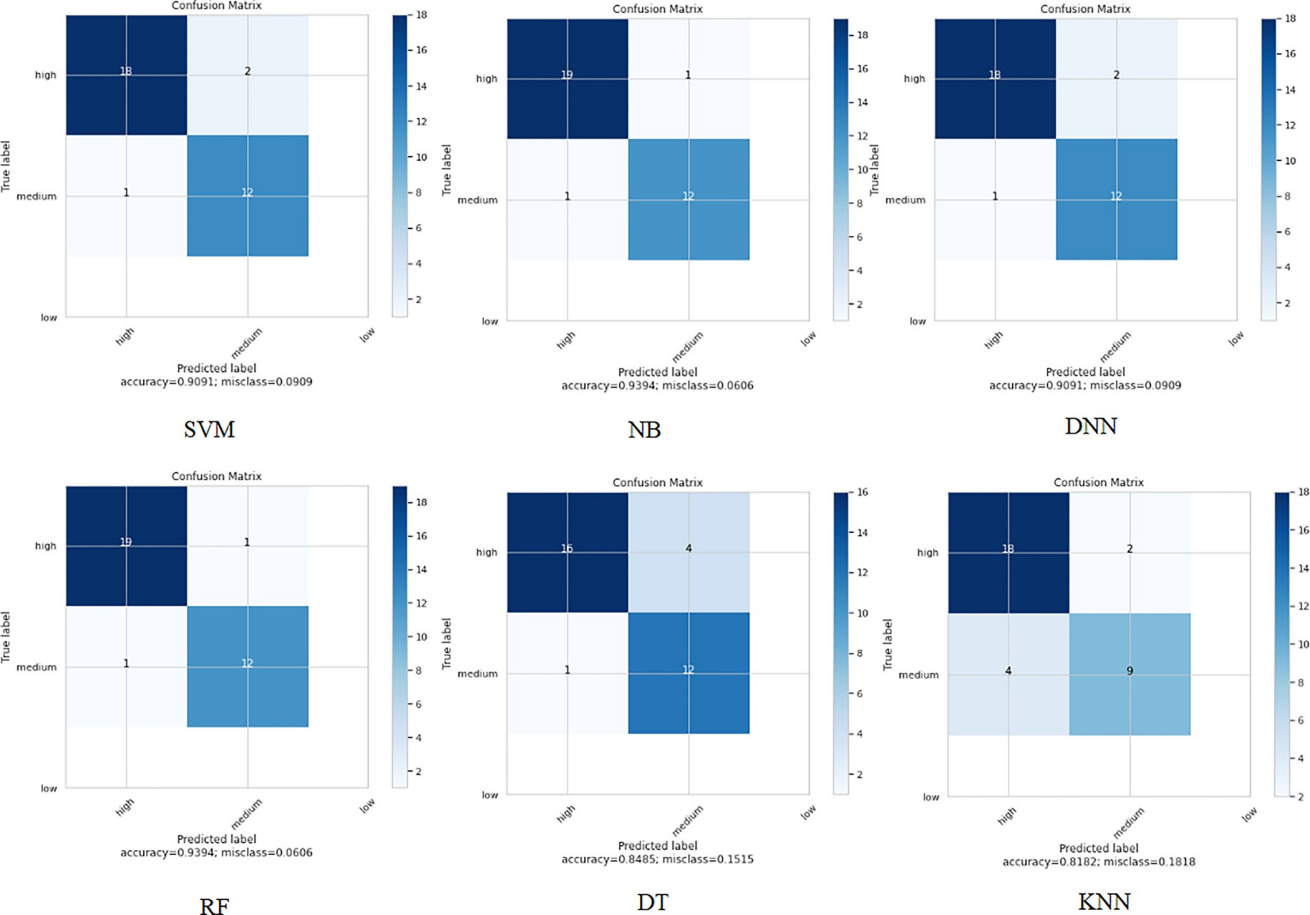
ML confusion matrix for the ML algorithms applied to Dataset 3.

**Fig 15:**
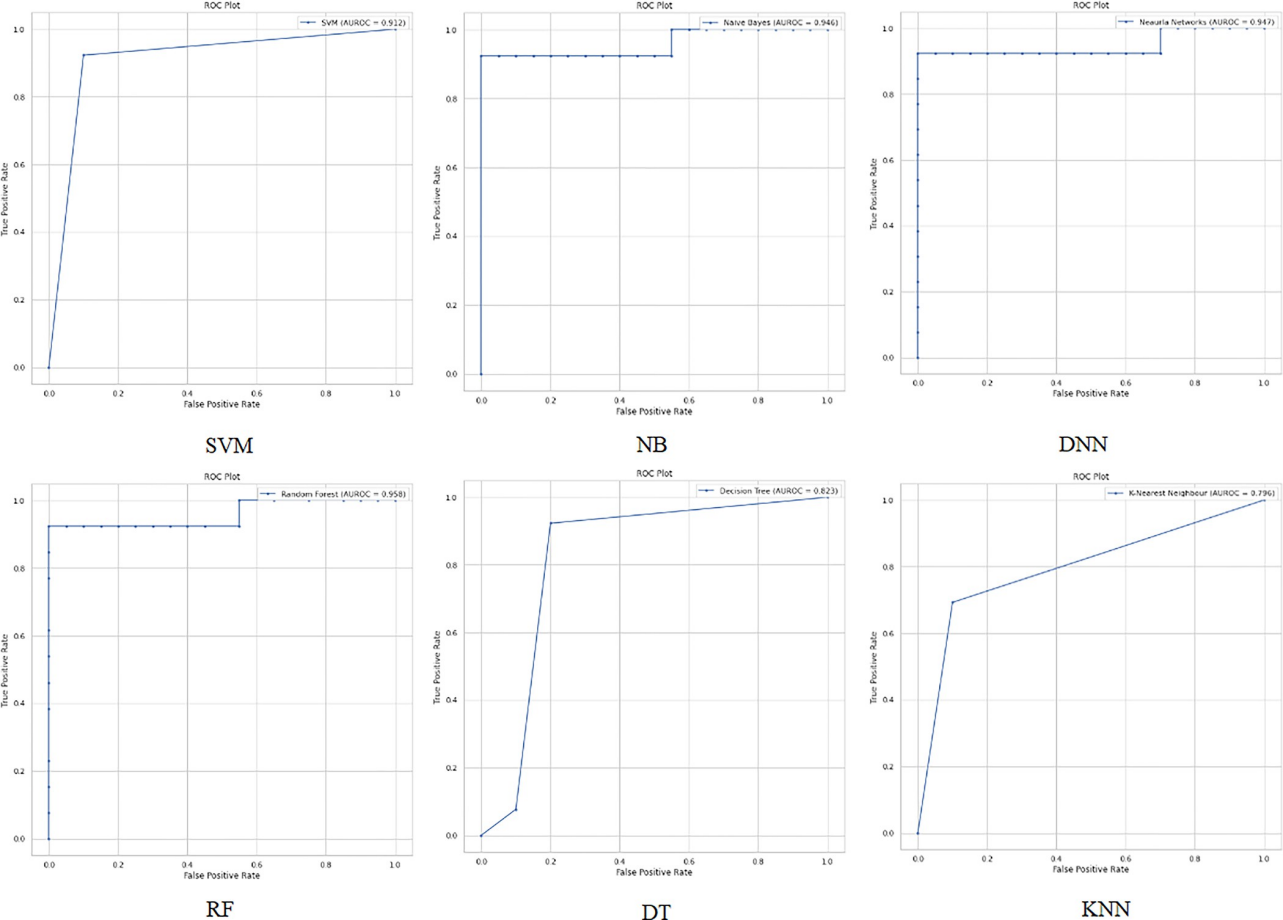
ROC area for the ML algorithms applied on Dataset 3.

**Fig 16:**
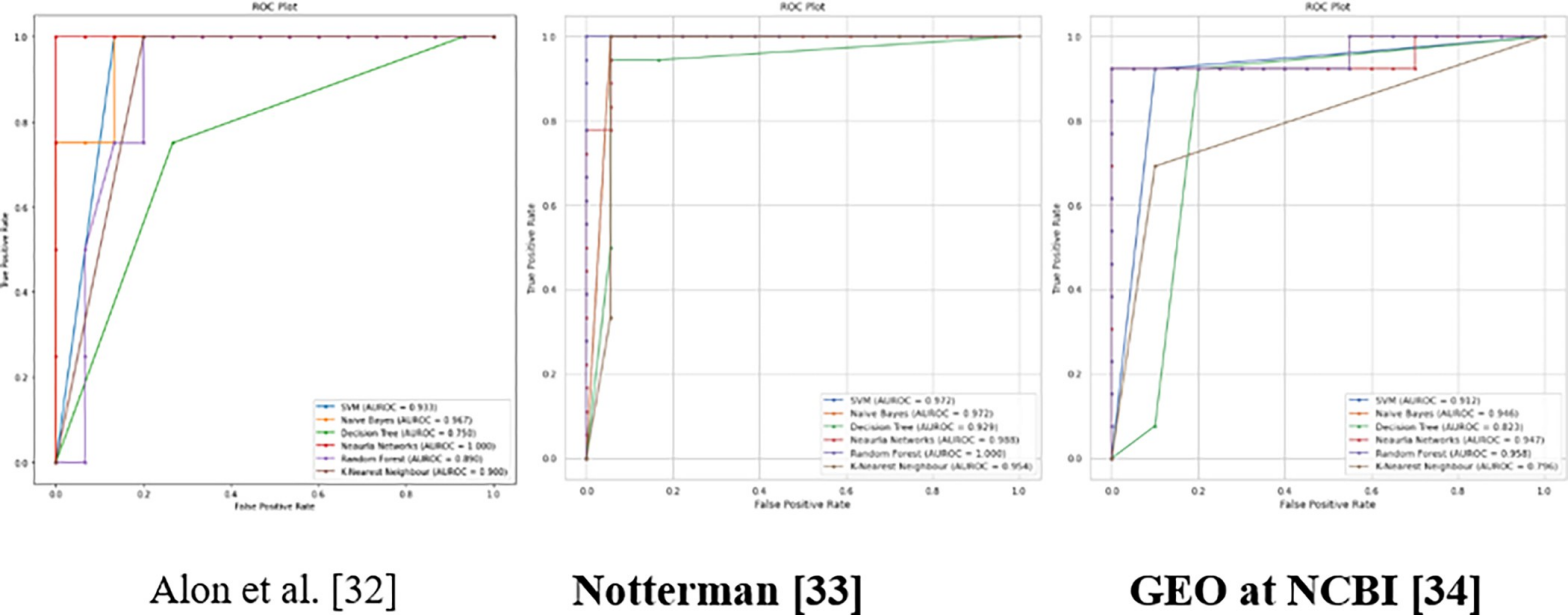
Summary of ROC area for the ML algorithms applied to all datasets.

When applying the proposed model to the third colon cancer dataset (GEO at NCBI) [47], the NB and RF algorithms show the highest classification accuracy of almost 94%. Meanwhile, DNN and SVM report promising accuracy results (91%). Figs 13 and 14 present the accuracy performance and confusion matrices, respectively, for the ML algorithms applied to this dataset, and Fig 15 illustrates the ROC measure for the classification of the machine learning algorithms applied to the same dataset. Finally, Fig 16 provides a summary of the performance. The RF, DNN and NB have a high area of the curve classifying the positive cases.

## 7. Evaluation

The performance of the trained models has been evaluated using various evaluation methods, such as the confusion matrix, which is used to compute the accuracy, recall, precision, F1-Score, and Area Under the Curve (AUC). The accuracy of the classification system is simply the ratio of the number of cases predicted correctly to the total number of cases. Recall, also known as sensitivity, is the ratio of the number of correctly predicted positive cases to all observations of the actual class. The precision measurement shows the ratio of the number of correct positive results to all positive results. The F1-score is calculated by applying weighted averages on precision and recall. Since we have imbalanced dataset, then F1-score is commonly more valuable than precision because it takes both false positives and false negatives into account [40]. Following are the equations to compute the evaluation metrics for performance assessment used in the paper.


$$Accuracy=\frac{TP+TN}{\left(TP+FN+FP+TN\right)}$$ (1)

$$Recall=\frac{TP}{\left(TP+FN\right)}$$ (2)

$$Precision=\frac{TP}{\left(TP+FP\right)}$$ (3)

$$F1-Score=2\times \frac{Precision\times Recall}{Precision+Recall}$$ (4)

### 7.1 Model evaluation using (IG + GA)

Since the datasets are either balanced and imbalanced, we used accuracy, recall, precision, F1-Score, and ROC metrics to assess the performance of the classification algorithms for the three datasets. These output measures are extracted from the confusion matrix that is used to evaluate the performance of the classifiers.

Tables 4–6 show results for datasets 1, 2 and 3 respectively. These results present the performance analysis for classifying the data using a variety of machine learning algorithms when applying the first phase, i.e., the multifilter hybrid feature selection (IG + GA) only. For dataset 1 (Alon et al. [37]), it was observed that SVM, KNN, and DNN showed good results with an accuracy of 89.5%, and an F-Measure of 0.933, 0.933, and 0.9 respectively. However, for dataset 2 (Notterman) [46], the classifiers of SVM, NB, DNN, RF, and KNN were observed to have good results with an accuracy of 97.2% for all of them, and an F1-Measure of 0.973. On the other hand, dataset 3 (GEO at NCBI) [47], the classifiers of NB, DNN, and RF had shown good results of almost 91%, with an F1-Measure of 0.923, 0.927, and 0.923 respectively.

**Table 4:** Phase 1 performance measures for Dataset 1.

	Dataset 1 Alon et al. [47]				
Classifier	Precision	Recall	F-Measure	ROC Area	Accuracy
**SVM**	0.667	1	0.8	0.933	89.5%
**NB**	0.5	1	0.667	0.917	78.9%
**DT**	0.429	0.75	0.545	0.75	73.7%
**DNN**	0.667	1	0.8	0.9	89.5%
**RF**	0.571	1	0.727	0.875	84.2%
**K-NN**	0.667	1	0.8	0.933	89.5%

**Table 5:** Phase 1 performance measures for Dataset 2.

	Dataset 2 Notterman [39]				
Classifier	Precision	Recall	F-Measure	ROC Area	Accuracy
**SVM**	0.947	1	0.973	0.946	97.2%
**NB**	0.947	1	0.973	0.972	97.2%
**DT**	0.944	0.944	0.944	0.929	94.4%
**DNN**	0.947	1	0.973	1	97.2%
**RF**	0.947	1	0.973	1	97.2%
**K-NN**	0.947	1	0.973	0.954	97.2%

**Table 6:** Phase 1 performance measures for Dataset 3.

	Dataset 3 GEO at NCBI [40]				
Classifier	Precision	Recall	F-Measure	ROC Area	Accuracy
**SVM**	0.857	0.9	0.878	0.835	84.8%
**NB**	0.947	0.9	0.923	0.958	90.9%
**DT**	0.941	0.8	0.865	0.823	84.8%
	0.905	0.95	0.927	0.923	90.9%
**RF**	0.947	0.9	0.923	0.965	90.9%
**K-NN**	0.857	0.9	0.878	0.835	84.8%

### 7.2 Model evaluation using (IG + GA) and (mRMR + PSO)

Here, we evaluate our model on the three datasets respectively, using the proposed algorithms (IG + GA) for feature extraction, and (mRMR + PSO) for pre-training selection.

Tables 7–9 report the performance results of our proposed colon cancer analytical model, for datasets 1, 2 and 3 respectively. The same ML algorithms (i.e., SVM, NB, DT, DNN, RF, K-NN) have been used to train the model and extract the predictive tumour genes, by using both experimental phases. For dataset 1 (Alon et al. [37]), it was observed that KNN, SVM, and DNN showed good performance with an accuracy of 84.2%, 89.5% and 94.7%, respectively, with best F1-measure (0.89) for DNN. However, for dataset 2 (Notterman) [46], the classifiers of SVM, NB, RF and DNN, were observed to have good results with an accuracy of 97.2% for all of them, and an F1-Measure of 0.973. For dataset 3 (GEO at NCBI) [47], the classifiers of NB and RF had the best performance accuracy (93.9%), with an F1-Measure of 0.95.

**Table 7:** Phase 2 performance measures for Dataset 1.

Classifier	Precision	Recall	F-Measure	ROC Area	Accuracy
**SVM**	0.667	1	0.8	0.933	89.5%
**NB**	0.4	1	0.571	0.967	68.4%
**DT**	0.43	0.75	0.55	0.75	73.7%
**DNN**	**0.80**	**1**	**0.89**	**1**	**94.7%**
**RF**	0.50	0.75	0.60	0.89	78.9%
**K-NN**	0.57	1	0.73	0.90	84.2%

**Table 8:** Phase 2 performance measures for Dataset 2.

Classifier	Precision	Recall	F-Measure	ROC Area	Accuracy
**SVM**	0.947	1	0.973	0.972	**97.2%**
**NB**	0.947	1	0.973	0.972	**97.2%**
**DT**	0.944	0.944	0.944	0.929	94.4%
**DNN**	0.944	0.944	0.889	0.988	94.4%
**RF**	0.947	1	0.973	1	**97.2%**
**K-NN**	0.947	1	0.973	0.954	**97.2%**

**Table 9:** Phase 2 performance measures for Dataset 3.

Classifier	Precision	Recall	F-Measure	ROC Area	Accuracy
**SVM**	0.947	0.9	0.923	0.912	90.9%
**NB**	0.95	0.95	0.95	0.946	**93.9%**
**DT**	0.941	0.8	0.865	0.823	84.8%
**DNN**	0.947	0.9	0.923	0.947	90.9%
**RF**	0.95	0.95	0.95	0.958	**93.9%**
**K-NN**	0.818	0.9	0.857	0.796	81.8%

Fig 16 displays the ROC value and the AUC for all the three datasets by applying the proposed model and the classifier algorithms. For the first colon cancer dataset (Alon et al.) [37], the DNN, and NB have the highest AUC with 1 and 0.967 respectively for classifying the positive cases. The second colon cancer dataset (Notterman) [46], RF, and DNN have the highest AUC of 1 and 0.988 respectively. Finally, for the third colon cancer dataset (GEO at NCBI) [47], RF and DNN has the highest area under the curve of 0.958 and 0.947 respectively. In summary, DNN and RF show promising classification and high-performance results compared to other classifiers on different colon cancer datasets which can be used to detect and classify tumor colon cancer tissues.

### 7.3 Comparative evaluation

Overall, the proposed two-stage multi-filter model is more accurate in prediction and the number of selected genes than previous reported models, as illustrated in Table 10. For example, our proposed model achieved 95% with dataset 1 for 22 genes; with dataset 2, it achieved 97% for 35 genes; and with dataset 3, it was 94% for 68 genes. The F-Score is a very good performance measure for all of them.

**Table 10:** Comparison of the proposed model, with others in the literature.

Reference	Accuracy (%)
***Dataset 1 [KentRidge dataset] [37]***	
Salem et al. [22]	85.48
Sreepada et al. [41]	87.5
Li et al. [35]	91.9
Rathore et al. [45]	91.94
Zhang & Cao [27]	92.01
Abdi et al. [34]	93.55
Shutao et al. [33]	93.6
**Our Proposed Model–HMLFSM**	**95.0**
***Dataset 2 [Notterman dataset] [46]***	
Rathore et al. [45]	88.89
Al Snousy et al. [48]	97.22
Rathore et al.[49]	97.22
**Our Proposed Model–HMLFSM**	**97.22**
***Dataset 3 [GEO at NCBI] [47]***	
**Our Proposed Model–HMLFSM**	**93.9**

Our results clearly demonstrate that our proposed two-stage multi-filter model outperforms all of the previously reported models in Table 10 in terms of accuracy. For example, with dataset 1, our proposed model achieved an accuracy of 95%, which is higher than all other models, including the highest accuracy of 93.6% reported by Shutao et al. [33]. Similarly, with dataset 2, our proposed model achieved an accuracy of 97%, which is the same as the highest accuracy achieved by Rathore et al [45], but our model selected fewer genes [35] compared to Rathore et al. (almost 60). With dataset 3, our proposed model achieved an accuracy of 93.9%, which is considered a very good accuracy. However, no other studies had applied the same dataset in previous research. The results show that our proposed model can achieve higher accuracy with fewer selected genes, making it more efficient and cost-effective for gene selection in microarray data analysis. Therefore, our proposed model has significant advantages over existing state-of-the-art methods.

## 8. Conclusion and future work

In this research, we have designed, implemented, and evaluated an analytical framework model for gene tissue classification using ML models. We have adopted a predictive strategy that copes with the nature of the studied domain. The human genome is known to be highly dense and diverse, which make the analytical features hard to be filtered and selected. Hence, the random changing expressions of cancer genes and their unexpected growth make the analytical task even more challenging.

Our novel hybrid multifilter IG-GA feature extractor followed by mRMR-PSO have been tested on three different colon cancer related datasets; to the author’s best knowledge, this paper is the first one which combine three datasets for colon cancer gene analytics. We have significantly excluded more than 99% of initial input features with a hybrid application. The genetic information remained preserved through the selected features, which can be seen throughout the rapid surge of the accuracy performance (95%, ~97% and ~94% accuracies for the three datasets 1, 2 and 3 respectively). Our framework model–HMLFSM could be adapted in medical practice such as the following.

- Cancer gene therapy selection, by considering the patient’s immune responses [50] or prediction of response.
- Cancer gene tissue expression, which can be generalized to other cases, e.g., blood, kidney, etc. A more abstract data extractor could be more suitable to ensure the domain adaptability rather than microarrays.
- In addition to possible application of other learning models, e.g., RNNs, CNNs [51] to optimize the gene tissue representation, and the wider approximation of activation functions.

## Supporting information

S1 Dataset (RAR)

S1 File (PDF)

## References

[1] Foster KR, Koprowski R, Skufca JD. Machine learning, medical diagnosis, and biomedical engineering research—commentary. Biomed Eng Online. (2014) 5;13:94. doi: 10.1186/1475-925X-13-94 ; PMCID: PMC4105825. 24998888 PMC4105825

[2] Sengupta D. Chapter 23—Machine learning in precision medicine, (2021), In Intelligent Data-Centric Systems, Machine Learning, Big Data, and IoT for Medical Informatics, Academic Press, pp. 405–419, 10.1016/B978-0-12-821777-1.00013-6.

[3] Saporetti C.M, Fonseca D.L, Oliveira L.C, Pereira E, Goliatt L. Hybrid machine learning, models for estimating total organic carbon from mineral constituents in core samples of shale gas fields. (2022). *Marine and Petroleum Geology*. Vol 143.

[4] D’Hondt E, Ashby T.J, Chakroun I. et al. Identifying and evaluating barriers for the implementation of machine learning in the intensive care unit. (2022). Communications Medicine 2, 162. doi: 10.1038/s43856-022-00225-1 36543940 PMC9768782

[5] Elemento O, Leslie C, Lundin J. et al. Artificial intelligence in cancer research, diagnosis and therapy. *Nat Rev Cancer* 21, 747–752 (2021). doi: 10.1038/s41568-021-00399-1 34535775

[6] Sharma A., Rani R. A Systematic Review of Applications of Machine Learning in Cancer Prediction and Diagnosis. *Archives of Computational Methods in Engineering* 28, 4875–4896 (2021). 10.1007/s11831-021-09556-z

[7] Kourou K, Exarchos TP, Exarchos KP, Karamouzis MV, Fotiadis DI. Machine learning applications in cancer prognosis and prediction. Comput Struct Biotechnol J. 2014 Nov 15;13:8–17. doi: 10.1016/j.csbj.2014.11.005 ; PMCID: PMC4348437. 25750696 PMC4348437

[8] Schroeder B. Using machine learning to identify undiagnosable cancers. (2022). Koch Institute. Using machine learning to identify undiagnosable cancers | MIT News | Massachusetts Institute of Technology. Accessed on 10/12/2022

[9] Mangasarian Olvi & Street Nick & Wolberg William. (1970). Breast Cancer Diagnosis and Prognosis Via Linear Programming. Operations Research. 43. doi: 10.1287/opre.43.4.570

[10] Huang M-W, Chen C-W, Lin W-C, Ke S-W, Tsai C-F (2017) SVM and SVM Ensembles in Breast Cancer Prediction. PLoS ONE 12(1): e0161501. doi: 10.1371/journal.pone.0161501 28060807 PMC5217832

[11] Tufail AB, Ma YK, Kaabar MKA, Martínez F, Junejo AR, Ullah I, Khan R. Deep Learning in Cancer Diagnosis and Prognosis Prediction: A Minireview on Challenges, Recent Trends, and Future Directions. Comput Math Methods Med. 2021 Oct 31;2021:9025470. doi: 10.1155/2021/9025470 ; PMCID: PMC8572604. 34754327 PMC8572604

[12] Tiwari M. Microarrays and cancer diagnosis. J Cancer Res Ther. 2012 Jan-Mar;8(1):3–10. doi: 10.4103/0973-1482.95166 22531505

[13] Mohr S, Leikauf G.D, Keith G, Rihn B.H. Microarrays as Cancer Keys: An Array of Possibilities. (2002). Journal of Clinical Oncology. doi: 10.1200/JCO.2002.12.073 12118031

[14] Shafi A.S.M., Molla M.M.I., Jui J.J. et al. “Detection of colon cancer based on microarray Dataset using machine learning as a feature selection and classification techniques”, 2020, SN Appl. Sci. 2, 1243. doi: 10.1007/s42452-020-3051-2

[15] Shehab M., Abualigah L., Shambour Q., Abu-Hashem M. A., Khaled Yousef Shambour M., Izzat Alsalibi A., Gandomi A. H., “Machine learning in medical applications: A review of state-of-the-art Methods”, 2022, Computers in Biology and Medicine, Volume 145, 105458, ISSN 0010-4825, doi: 10.1016/j.compbiomed.2022.105458 35364311

[16] Lu Y, Han J, Cancer classification using gene expression data, Information Systems 28 (4) (June 2003) 243–268. 10.1016/S0306-4379(02)00072-8

[17] Al-Rajab M, Lu J, Xu Q (2021) A framework model using multifilter feature selection to enhance colon cancer classification. PLoS ONE 16(4): e0249094. doi: 10.1371/journal.pone.0249094 33861766 PMC8691854

[18] Groen AK, The pros and cons of gene expression analysis by microarrays. (2001). Volume 35, Issue 2, pp 295–296. doi: 10.1016/s0168-8278(01)00156-8 11580155

[19] ÖZCAN S˙IM SEK, ÖZGÜR A, GÜRGEN F. Biology Direct. (2021). 16:7. 10.1186/s13062-020-00290-3 33557857 PMC7869482

[20] Touchanti I.T, Ezzazi M.E. & Maser S. “A 2-stages feature selection framework for colon cancer classification using SVM," 2022 International Conference on Intelligent Systems and Computer Vision (ISCV), (2022), pp. 1–5, doi: 10.1109/ISCV54655.2022.9806115

[21] Mufassirin MMM & Ragel RG, "A Novel Filter-Wrapper Based Feature Selection Approach For Cancer Data Classification,” 2018 IEEE International Conference on Information and Automation for Sustainability (ICIAfS), 2018, pp. 1–6, doi: 10.1109/ICIAFS.2018.8913362

[22] Salem Hanaa, Attiya Gamal, Nawal El-Fishawy. (January 2017). “Classification of human cancer diseases by gene expression profiles”, Applied Soft Computing, Volume 50, Pages 124–134, ISSN 1568-4946, 10.1016/j.asoc.2016.11.026

[23] Pashaei E.”Gene Selection for Cancer Classification using a New Hybrid of Binary Black Hole Algorithm”, 2020 28^th^ Signal Processing and Communications Applications Conference (SIU), 2020, pp. 1–4, doi: 10.1109/SIU49456.2020.9302351

[24] Nazari E, Aghemiri M, Avan A, Mehrabian A & Tabesh H. (2021). Machine learning approaches for classification of colorectal cancer with and without feature selection method on Microarray Data. Gene Reports. 25. 101419. doi: 10.1016/j.genrep.2021.101419

[25] Talukder MA, Islam MM, Uddin MA, Arnisha A, Hasan KF, Moni MA, “Machine learning-based lung and colon cancer detection using Deep feature extraction and ensemble learning”, (2022). Expert Systems with Applications, Volume 205, 117695, ISSN 0957-4174, 10.1016/j.eswa.2022.117695.

[26] Cahnyaningrum KA & Astuti W, “Microarray Gene Expression Classification for Cancer Detection using Artificial Neural Networks and Genetic Algorithm Hybrid Intelligence”, (2020). International Conference on Data Science and Its Applications (ICoDSA), 2020, pp. 1–7, doi: 10.1109/ICoDSA50139.2020.9213051

[27] Zhang B, Cao P (2019). “Classification of high dimensional biomedical data based on feature selection using redundant removal”. PLOS ONE, Volume 14, Issue: 4: e0214406. doi: 10.1371/journal.pone.0214406 30964868 PMC6456288

[28] Houssein Essam H., Diaa Salama Abdelminaam Hager N. Hassan, Mustafa M. Al-Sayed, and Emad Nabil. "A hybrid barnacles mating optimizer algorithm with support vector machines for gene selection of microarray cancer classification." IEEE Access 9 (2021): 64895–64905

[29] Houssein, Essam H., Zainab Abohashima, Mohamed Elhoseny, and Waleed M. Mohamed. "An efficient binary harris hawks optimization based on quantum SVM for cancer classification tasks." In The 2nd International Conference on Distributed Sensing and Intelligent Systems (ICDSIS 2021), vol. 2021, pp. 247–258. IET, 2021.

[30] Houssein Essam H., Hager N. Hassan, Mustafa M. Al-Sayed, and Emad Nabil. "Gene selection for microarray cancer classification based on manta rays foraging optimization and support vector machines." Arabian Journal for Science and Engineering (2022): 1–18.

[31] Shutao LW2, Xiaoyan XH, (2008). “Gene selection using genetic algorithm and support vector machines”, Soft Comput. 12 (7) 693–698, 10.1007/s00500-007-0251-2

[32] Mohamad M, Deris S, Illias R, (2005). “A hybrid of genetic algorithm and support vector Machine for features selection and classification of gene expression microarray”, International Journal of Computational Intelligence and its Applications 5, pp. 91–107. 10.1142/S1469026805001465

[33] Shutao L2, Xixian W, Xiaoyan H. (2008). “Gene selection using genetic algorithm and support vector machines”, Soft Computing 12 (7) 693–698, 10.1007/s00500-007-0251-2

[34] Abdi MJ, Hosseini SM & Rezghi M. (2012). “A Novel Weighted Support Vector Machine Based on Particle Swarm Optimization for Gene Selection and Tumor Classification”, COMPUTATIONAL and Mathematical Methods in Medicine, vol. 2012, Article ID 320698, 7 pages. doi: 10.1155/2012/320698 22924059 PMC3424529

[35] Li S, Wu X & Tan ML. (2008). “Gene selection using hybrid particle swarm optimization and genetic algorithm”, Soft Comput. 12, 11 (September 2008), pp. 1039–1048. doi: http%3A//doi.org/10.1007/s00500-007-0272-x

[36] Fahami MA, Roshanzamir M, Hoseini Izadi N, Keyvani V, Alizadehsani R, Detection of effective genes in colon cancer: A machine learning approach, Informatics in Medicine Unlocked, Volume 24, 2021, 100605, ISSN 2352-9148, 10.1016/j.imu.2021.100605.

[37] Alon U, Barkai N, Notterman DA, Gish K, Ybarra S, Mack D, Levine AJ (1999) Broad patterns of gene expression revealed by clustering analysis of tumor and normal colon tissues probed by oligonucleotide arrays. Proc Natl Acad Sci USA 96:6745–6750 doi: 10.1073/pnas.96.12.6745 10359783 PMC21986

[38] Eibe F, Hall MA, and Witten IH (2016). The WEKA Workbench. Online Appendix for "Data Mining: Practical Machine Learning Tools and Techniques", Morgan Kaufmann, Fourth Edition, 2016

[39] Dash S. and Patra, B., (2012). “BIOCOMP Study of Classification Accuracy of Microarray Data for Cancer Classification using Hybrid, Wrapper and Filter Feature Selection Method”, In Proceedings of the International Conference on Bioinformatics & Computational Biology (BIOCOMP) (p. 268). The Steering Committee of The World Congress in Computer Science, Computer Engineering and Applied Computing (WorldComp).

[40] Ali E, El-K Aouatif A, Abdeljalil EA & Driss A. (2011). “A two-Stage gene selection scheme utilizing MRMR filter and GA wrapper”, Knowledge and Information Systems. 26. 487–500. 10.1007/s10115-010-0288-x.

[41] Sreepada RS, Vipsita S, Mohapatra P, (2015). "An efficient approach for microarray data Classification using filter wrapper hybrid approach”, IEEE International Advance Computing Conference (IACC), Banglore, 2015, pp. 263–267. 10.1109/IADCC.2015.7154710

[42] Yeh J-Y, Wu T-S, Wu M-C & Chang D-M, (Nov. 2007). "Applying Data Mining Techniques For Cancer Classification from Gene Expression Data”, in International Conference on Convergence Information Technology, pp.703,708, 21–23. doi: 10.1109/ICCIT.2007.153

[43] Garcia-Nieto ELJ, Jourdan L & Talbi E. (2007). "Gene Selection In Cancer Classification using PSO/SVM and GA/SVM Hybrid Algorithms", IN IEEE Congress On Evolutionary Computation, 2007. CEC 2007. PP.284,290. doi: 10.1109/CEC.2007.4424483

[44] Akadi El, Ali & Aouatif Amine& Abdeljalil El Ouardighi & Driss Aboutajdine. (2011). “A two-stage gene selection scheme utilizing MRMR filter and GA wrapper”, Knowledge and Information Systems. 26. 487–500. doi: http%3A//doi.org/10.1007/s10115-010-0288-x

[45] Rathore S^3^, Iftikhar MA & Hussain M, "A novel approach for automatic gene selection and Classification of gene based colon cancer datasets,” *2014 International Conference on Emerging Technologies (ICET)*, 2014, pp. 42–47, doi: 10.1109/ICET.2014.7021014

[46] Notterman DA, Alon U, Sierk AJ, Levine AJ. (2001). “Transcriptional gene expression profiles of colorectal adenoma, adenocarcinoma, and normal tissue examined by oligonucleotide arrays”, Cancer Research., Volume. 61, no. 7, Pages. 3124–3130. 11306497

[47] Schetter AJ, Ryan BM, Harris CC, “GEO Accession viewer,” Nih.gov. [Online]. Available: https://www.ncbi.nlm.nih.gov/geo/query/acc.cgi. [Accessed: 30-Jul-2022].

[48] Al Snousy MB, El-Deeb HM, Badran K, Al Khlil IA. “Suite of decision tree-based classification algorithms on cancer gene expression data”, (2011), Egyptian Informatics Journal, Volume 12, Issue 2, Pages 73–82, ISSN 1110-8665, 10.1016/j.eij.2011.04.003.

[49] Rathore S4, Hussain M, & Khan A. (2014). “GECC: gene expression based ensemble Classification of colon samples”, IEEE/ACM Transactions on Computational Biology and Bioinformatics (TCBB), 11(6), 1131–1145. doi: 10.1109/TCBB.2014.2344655 26357050

[50] Rafique R, Islam SMR, Kazi JU. “Machine learning in the prediction of cancer therapy”. Computational and Structural Biotechnology Journal 2021 Jul 8;19:4003–4017. doi: 10.1016/j.csbj.2021.07.003 ; PMCID: PMComputational and Structural Biotechnology JournalC8321893 34377366 PMC8321893

[51] Ahmed O, Brifcani A. “Gene Expression Classification Based on Deep Learning”. (2020). 4th Scientific International Conference–Najaf–IRAQ (4th -SICN-2019). 10.1109/SICN47020.2019.9019357

