# Supplementary material for: A hybrid machine learning feature selection model—HMLFSM to enhance gene classification applied to multiple colon cancers dataset
Source: PLoS One. 2023 Nov 2;18(11):e0286791. doi: 10.1371/journal.pone.0286791 (PMC10621932; doi:10.1371/journal.pone.0286791)
Supplement: S1 File — (PDF) [file pone.0286791.s002.pdf]

```
# Importing the required libraries
import pandas as pd
import numpy as np

#Visualization libraries
import matplotlib.pyplot as plt
from matplotlib import pyplot
import matplotlib.colors as colors
import seaborn as sns

# Importing the library for splitting
from sklearn.metrics import accuracy_score
from sklearn.model_selection import train_test_split
from sklearn.model_selection import cross_val_score

# Importing machine learning models
from sklearn import svm
# from sklearn.svm import SVR
# from sklearn.linear_model import LinearRegression
from sklearn.svm import SVC
from sklearn.linear_model import LogisticRegression
#from fbprophet import Prophet
from sklearn.tree import DecisionTreeClassifier
from sklearn.ensemble import RandomForestClassifier
from sklearn.naive_bayes import GaussianNB
from sklearn.neighbors import KNeighborsClassifier
from sklearn.neural_network import MLPClassifier
from sklearn.gaussian_process import GaussianProcessClassifier
from sklearn.gaussian_process.kernels import RBF
from sklearn.ensemble import AdaBoostClassifier


from sklearn.metrics import plot_confusion_matrix
from sklearn.metrics import classification_report
from sklearn.discriminant_analysis import QuadraticDiscriminantAnalysis
from sklearn.metrics import precision_score
from sklearn.metrics import recall_score
from sklearn.metrics import f1_score
```

```
from google.colab.data_table import DataTable
DataTable.max_columns = 30
```

```
df=pd.read_csv('/content/NottermanMurad2.csv')
```

```
from google.colab import data_table
data_table.enable_dataframe_formatter()

df.head()
```

```
Warning: Total number of columns (1968) exceeds max_columns (30). Falling back to pandas display.
```

	attribute1	attribute2	attribute3	attribute4	attribute5	attribute6	attribute7	attribute8	attribute9	attribute10
**0**	26	18	-7	70	65	203	254	28	72	92
**1**	13	13	-7	138	64	260	327	81	89	84
**2**	13	11	-6	73	79	305	355	60	85	115
**3**	9	28	-17	27	62	166	230	18	55	69
**4**	2	6	10	125	86	362	252	73	54	115

5 rows × 1968 columns

```
#highlight the dataframe
df.style.background_gradient(cmap='Reds')
```

	Unnamed: 0	1007_s_at	1053_at	117_at	121_at	1255_g_at	1294_at	1316_at	1320_at	1405_i_at	1431_at	1438_
**0**	GSM1092909	11.762200	6.295420	5.556600	7.810900	5.242490	9.162200	5.749710	5.908990	7.645400	5.267950	6.1035
**1**	GSM1092910	10.578500	6.774510	5.585860	7.667290	5.218540	7.738650	5.971870	5.523960	6.876460	5.506460	5.7480
**2**	GSM1092911	10.882800	6.302810	5.615780	8.431180	5.332010	8.726610	6.566930	5.679540	7.695650	5.668340	5.5520
**3**	GSM1092912	11.233000	8.188850	6.095700	7.812320	5.302530	8.266050	5.693510	5.798420	7.642480	5.645310	10.1422
**4**	GSM1092913	10.055700	6.274960	5.998640	7.528460	5.302740	8.740450	6.336410	5.811550	8.514290	5.293940	5.6767
**5**	GSM1092914	8.719810	7.239630	6.774030	7.626690	5.193170	8.289320	6.184960	5.691580	7.151540	5.652880	5.5107
**6**	GSM1092915	10.103200	6.323910	5.622920	8.141570	5.399200	9.186070	6.621930	5.694750	7.473520	5.506110	5.7554
**7**	GSM1092916	10.336100	6.681810	5.627540	8.254350	5.370890	9.527720	6.594560	5.750010	7.697870	5.833060	6.0811

```
from pandas.plotting import scatter_matrix
scatter_matrix(df, figsize=(30, 40))
pyplot.show()
```

```
#Drop index, city, state
df['class'].unique()
```

```
array(['positive', 'negative'], dtype=object)
```

```
df.isnull().values.any()
```

```
False
```

```
df.isnull().sum()
```

```
attribute1       0
attribute2       0
attribute3       0
attribute4       0
attribute5       0
                ..
attribute1964    0
attribute1965    0
attribute1966    0
attribute1967    0
class            0
Length: 1968, dtype: int64
```

```
df.info()
```

```
<class 'pandas.core.frame.DataFrame'>
RangeIndex: 36 entries, 0 to 35
Columns: 1968 entries, attribute1 to class
dtypes: int64(1967), object(1)
memory usage: 553.6+ KB
```

```
df['class'].value_counts()
```

```
positive    18
negative    18
Name: class, dtype: int64
```

```
sns.countplot(df['class'],label = 'count')
```

```
/usr/local/lib/python3.7/dist-packages/seaborn/_decorators.py:43: FutureWarning: Pass the following variable as a keyword a
  FutureWarning
<matplotlib.axes._subplots.AxesSubplot at 0x7f9ae6d5e1d0>
```

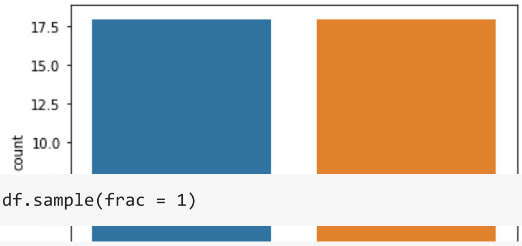


```
df = df.sample(frac = 1)
```

```
df.head(10)
```

```
Warning: Total number of columns (1968) exceeds max_columns (30). Falling back to pandas display.
```

	attribute1	attribute2	attribute3	attribute4	attribute5	attribute6	attribute7	attribute8	attribute9	attribute10
**2**	13	11	-6	73	79	305	355	60	85	115
**19**	50	117	19	28	75	233	210	97	138	117
**34**	43	81	21	23	105	149	101	83	48	126
**10**	15	8	-6	89	107	217	294	38	57	104
**0**	26	18	-7	70	65	203	254	28	72	92
**6**	20	26	-20	24	99	217	163	20	70	113
**17**	29	16	-17	175	84	180	242	48	72	58
**32**	42	85	-3	24	54	104	84	29	143	72
**14**	4	14	-16	58	96	178	157	21	69	63
**4**	2	6	10	125	86	362	252	73	54	115

10 rows × 1968 columns

```
df.columns
```

```
Index(['Unnamed: 0', '1007_s_at', '1053_at', '117_at', '121_at', '1255_g_at',
       '1294_at', '1316_at', '1320_at', '1405_i_at',
       ...
       'AFFX-r2-Ec-bioD-5_at', 'AFFX-r2-P1-cre-3_at', 'AFFX-r2-P1-cre-5_at',
       'AFFX-ThrX-3_at', 'AFFX-ThrX-5_at', 'AFFX-ThrX-M_at', 'AFFX-TrpnX-3_at',
       'AFFX-TrpnX-5_at', 'AFFX-TrpnX-M_at', 'CLASS'],
      dtype='object', length=22279)
```

```
df.describe()
```

```
Warning: Total number of columns (1967) exceeds max_columns (30). Falling back to pandas display.
```

	attribute1	attribute2	attribute3	attribute4	attribute5	attribute6	attribute7	attribute8	attribute9	attribute
**count**	36.000000	36.000000	36.000000	36.000000	36.000000	36.000000	36.000000	36.000000	36.000000	36.000
**mean**	31.166667	62.583333	-2.777778	51.055556	93.000000	198.972222	180.888889	47.277778	78.083333	100.750
**std**	20.487627	48.728622	14.420444	37.011152	26.398052	62.525646	79.002632	26.601408	29.418532	30.505
**min**	2.000000	6.000000	-22.000000	8.000000	34.000000	104.000000	56.000000	7.000000	32.000000	49.000
**25%**	15.750000	17.500000	-14.250000	24.750000	75.750000	157.250000	102.750000	27.500000	55.000000	72.000
**50%**	25.000000	48.500000	-6.000000	41.000000	88.500000	178.500000	177.500000	48.500000	71.500000	96.000
**75%**	46.250000	97.250000	8.500000	59.750000	106.250000	219.750000	236.500000	61.250000	93.500000	117.750
**max**	79.000000	206.000000	42.000000	175.000000	158.000000	368.000000	355.000000	112.000000	143.000000	156.000

8 rows × 1967 columns

```
print(X, Y)
```

```
[[11.7622   6.29542  5.5566  ...  5.50055  5.01456  5.04927]
 [10.5785   6.77451  5.58586 ...  5.52497  5.11313  5.05536]
 [10.8828   6.30281  5.61578 ...  5.59045  5.14005  5.19297]
 ...
 [10.0129   6.51293  6.32736 ...  5.23979  5.10725  4.99928]
 [10.9572   5.97877  5.79387 ...  4.93236  5.03841  5.42336]
 [11.4878   7.6279   6.07889 ...  4.73553  5.08259  5.04053]] ['tissue: adjacent nontumor' 'tissue: Tumor' 'tissue: adjacent nontum
 'tissue: Tumor' 'tissue: adjacent nontumor' 'tissue: Tumor'
 'tissue: Tumor' 'tissue: adjacent nontumor' 'tissue: adjacent nontumor'
 'tissue: Tumor' 'tissue: Tumor' 'tissue: adjacent nontumor'
```

```
 'tissue: Tumor' 'tissue: adjacent nontumor' 'tissue: Tumor'
 'tissue: adjacent nontumor' 'tissue: adjacent nontumor' 'tissue: Tumor'
 'tissue: Tumor' 'tissue: adjacent nontumor' 'tissue: adjacent nontumor'
 'tissue: Tumor' 'tissue: adjacent nontumor' 'tissue: Tumor'
 'tissue: adjacent nontumor' 'tissue: adjacent nontumor' 'tissue: Tumor'
 'tissue: adjacent nontumor' 'tissue: adjacent nontumor' 'tissue: Tumor'
 'tissue: adjacent nontumor' 'tissue: Tumor' 'tissue: adjacent nontumor'
 'tissue: Tumor' 'tissue: Tumor' 'tissue: adjacent nontumor'
 'tissue: adjacent nontumor' 'tissue: adjacent nontumor' 'tissue: Tumor'
 'tissue: adjacent nontumor' 'tissue: Tumor' 'tissue: Tumor'
 'tissue: Tumor' 'tissue: Tumor' 'tissue: adjacent nontumor'
 'tissue: Tumor' 'tissue: Tumor' 'tissue: adjacent nontumor'
 'tissue: Tumor' 'tissue: Tumor' 'tissue: adjacent nontumor'
 'tissue: Tumor' 'tissue: adjacent nontumor' 'tissue: adjacent nontumor'
 'tissue: Tumor' 'tissue: adjacent nontumor' 'tissue: Tumor'
 'tissue: adjacent nontumor' 'tissue: Tumor' 'tissue: adjacent nontumor'
 'tissue: Tumor' 'tissue: adjacent nontumor' 'tissue: Tumor'
 'tissue: Tumor' 'tissue: Tumor' 'tissue: adjacent nontumor'
 'tissue: adjacent nontumor' 'tissue: adjacent nontumor' 'tissue: Tumor'
 'tissue: adjacent nontumor' 'tissue: Tumor' 'tissue: Tumor'
 'tissue: adjacent nontumor' 'tissue: Tumor' 'tissue: adjacent nontumor'
 'tissue: adjacent nontumor' 'tissue: Tumor' 'tissue: adjacent nontumor'
 'tissue: Tumor' 'tissue: adjacent nontumor' 'tissue: Tumor'
 'tissue: Tumor' 'tissue: adjacent nontumor' 'tissue: Tumor'
 'tissue: Tumor' 'tissue: adjacent nontumor' 'tissue: adjacent nontumor'
 'tissue: Tumor' 'tissue: adjacent nontumor' 'tissue: Tumor'
 'tissue: adjacent nontumor' 'tissue: Tumor' 'tissue: adjacent nontumor'
 'tissue: Tumor' 'tissue: adjacent nontumor' 'tissue: Tumor'
 'tissue: adjacent nontumor' 'tissue: adjacent nontumor' 'tissue: Tumor'
 'tissue: adjacent nontumor' 'tissue: Tumor' 'tissue: adjacent nontumor'
 'tissue: Tumor' 'tissue: Tumor' 'tissue: adjacent nontumor'
 'tissue: adjacent nontumor' 'tissue: Tumor' 'tissue: adjacent nontumor'
 'tissue: Tumor' 'tissue: adjacent nontumor' 'tissue: Tumor']
```

Train Test Split

```
X_train, X_test, Y_train, Y_test = train_test_split(X, Y, test_size = 0.3, stratify = Y, random_state=1)
```

```
print(X_train.shape, X_test.shape, X.shape)
```

```
(77, 22276) (34, 22276) (111, 22276)
```

```
models = [LogisticRegression(max_iter=1000), knn, RandomForestClassifier(),GaussianNB(),DecisionTreeClassifier(), classifier, classifier2
```

```
def compare_models_cross_validation():
  for model in models:
    cv_score=cross_val_score(model, X, Y, cv = 5)
    mean_accuracy = sum(cv_score)/len(cv_score)
    mean_accuracy = mean_accuracy*100
    mean_accuracy = round(mean_accuracy, 2)

    print('Cross validation accuracies for', model,'= ' ,cv_score)
    print('Accuracy % of the', model, mean_accuracy)
    print('-------------------------------------')
```

```
import pandas as pd

from sklearn.neural_network import MLPClassifier
from sklearn.neighbors import KNeighborsClassifier
from sklearn.svm import SVC
from sklearn.gaussian_process import GaussianProcessClassifier
from sklearn.ensemble import GradientBoostingClassifier
from sklearn.gaussian_process.kernels import RBF
from sklearn.tree import DecisionTreeClassifier
from sklearn.ensemble import ExtraTreesClassifier
from sklearn.ensemble import RandomForestClassifier, AdaBoostClassifier
from sklearn.naive_bayes import GaussianNB
from sklearn.discriminant_analysis import QuadraticDiscriminantAnalysis
from sklearn.linear_model import SGDClassifier
```

```
from sklearn.model_selection import train_test_split
from sklearn.datasets import make_classification
```

```
#X, Y = make_classification()
df['CLASS']=df['CLASS'].map({'tissue: Tumor':1, 'tissue: adjacent nontumor': 0})
```

```
X_train, X_test, Y_train, Y_test = train_test_split(X, Y, test_size = 0.3, stratify = Y, random_state=1)
```

Accuracy

```
Accuracy = []
for name, clf in zip(names, classifiers):
    Accuracy.append(clf)
```

```
Accuracy
```

```
[90.9, 93.9, 84.8, 90.9, 93.9, 90.9]
```

```
from sklearn.metrics import recall_score

Recall = []
for name, clf in zip(names, classifiers):
    clf.fit(X_train, Y_train)
    X_test_prediction = clf.predict(X_test)
    recall = recall_score(X_test_prediction, Y_test, pos_label='tissue: Tumor')
    Recall.append(recall)
```

```
/usr/local/lib/python3.7/dist-packages/sklearn/metrics/_classification.py:1318: UndefinedMetricWarning: Recall is ill-defined and b
  _warn_prf(average, modifier, msg_start, len(result))
```

```
from sklearn.metrics import f1_score

F1_Score = []
for name, clf in zip(names, classifiers):
    clf.fit(X_train, Y_train)
    X_test_prediction = clf.predict(X_test)
    fscore = f1_score(X_test_prediction, Y_test, pos_label='tissue: Tumor')
    F1_Score.append(fscore)
```

Double-click (or enter) to edit

```
import pandas as pd
import seaborn as sns
```

```
df = pd.DataFrame()
df['name'] = names
df['Accuracy'] = Accuracy
df['Precision'] = Precision
df['Recall'] = Recall
df['F1 Score'] = F1_Score
df
```

```
#https://pandas.pydata.org/pandas-docs/stable/user_guide/style.html

cm = sns.light_palette("green", as_cmap=True)
s = df.style.background_gradient(cmap=cm)
s
```

	name	Accuracy	Precision	Recall	F1 Score
**0**	Nearest_Neighbors	0.852941	0.941176	0.800000	0.864865
**1**	SVM	0.882353	0.882353	0.882353	0.882353
**2**	Decision_Tree	0.852941	0.941176	0.722222	0.888889

```
Accuracy
```

```
[90.9, 93.9, 84.8, 90.9, 93.9, 90.9]
```

[illegible]

```
fig = plt.figure(figsize = (10, 10))
sns.set(style="whitegrid")
#ax = sns.barplot(y="name", x="Accuracy", palette = 'viridis')
sns.barplot(names,Accuracy)
#palette = 'magma'
#palette = 'hls'
#palette = 'deep'
#palette = 'rocket'
#palette = 'vlag'
#palette = 'tab10'
#palette = 'Paired'
#palette = 'mako'
#palette = 'crest'
#palette = 'flare'
```

```
/usr/local/lib/python3.7/dist-packages/seaborn/_decorators.py:43: FutureWarning: Pass the following variables as keyword ar
  FutureWarning
<matplotlib.axes._subplots.AxesSubplot at 0x7f92afc68050>
```

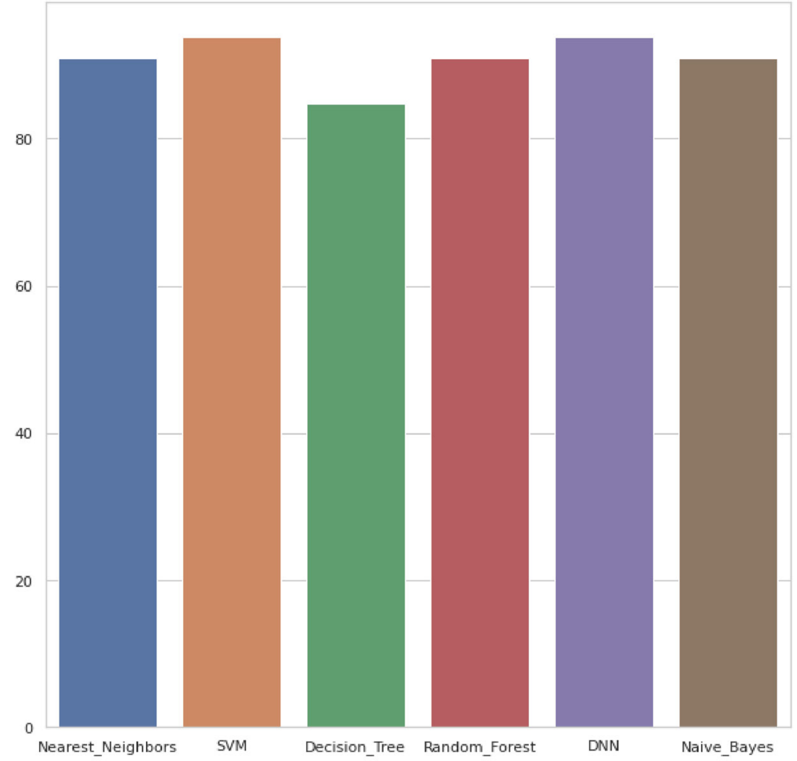


```
fig = plt.figure(figsize = (25, 10))
ax = fig.add_axes([0,0,1,1])
ax.bar(names,Accuracy)

plt.xlabel("Classification Algorithms")
plt.ylabel("Accuracy")
plt.title("Classification Accuracy Results")
plt.show()
```

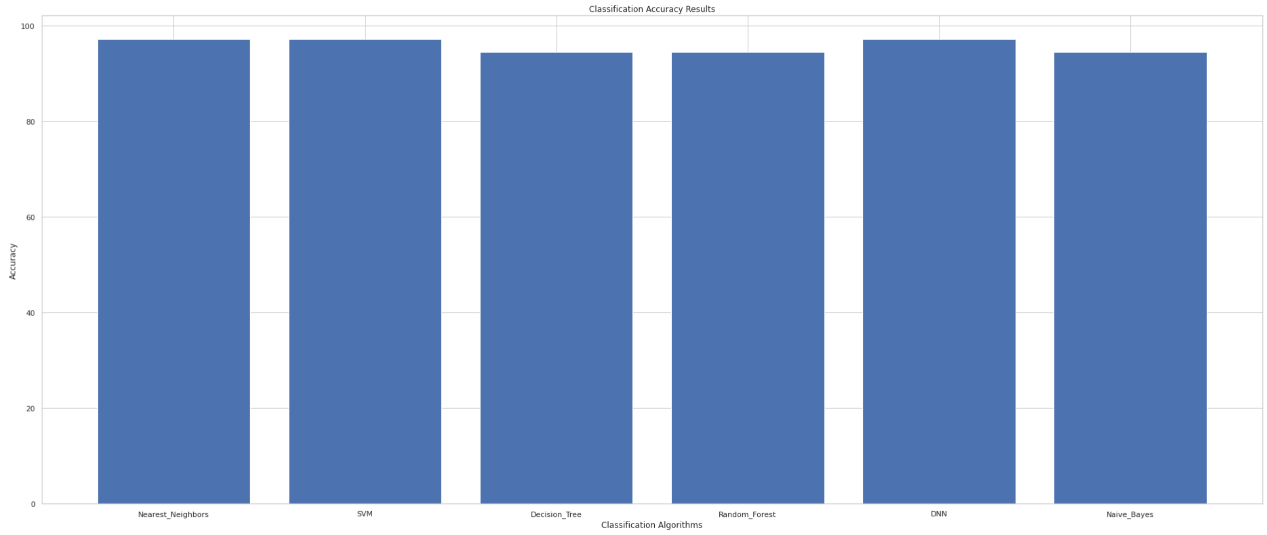


```
fig = plt.figure(figsize = (10, 10))
ax = fig.add_axes([0,0,1,1])
ax.barh(names,Accuracy)
plt.grid(color='#95a5a6', linestyle='--', linewidth=2, axis='y', alpha=0.7)

plt.xlabel("Accuracy")
plt.ylabel("Classification Algorithms")
plt.title("Classification Accuracy Results")
plt.show()
```

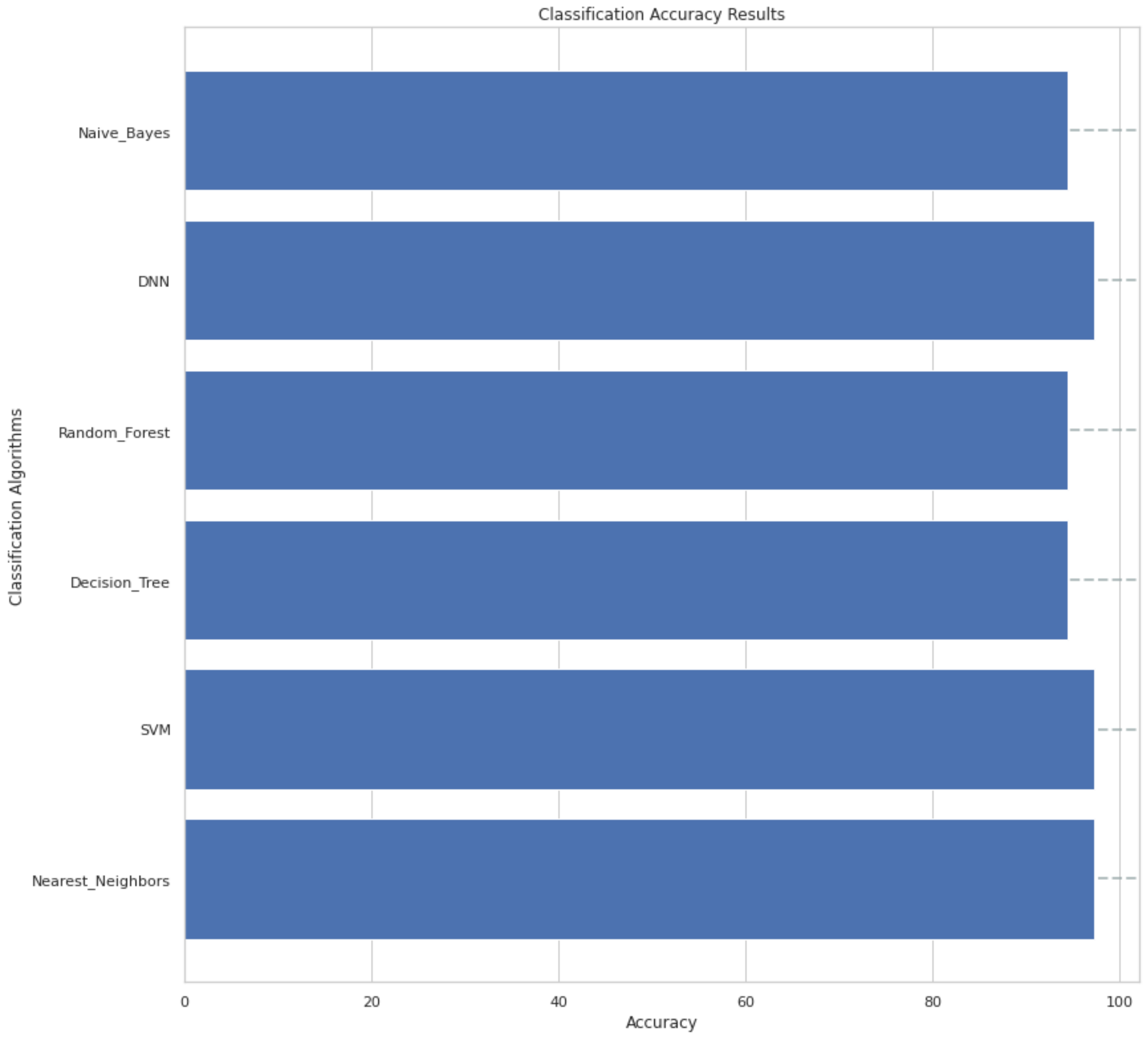

```
#SVM
TP = 18
FP = 2
FN = 1
TN = 12

svmAcc = (TP + TN) / (TP + TN + FN + FP)
print('SVM Testing Accuracy = ', svmAcc, '\n')
```

```
SVM Testing Accuracy =  0.9090909090909091
```

```
plot_confusion_matrix(cm           = np.array([[ 18,  2],
                                               [  1,  12,  ]]),
                      normalize    = False,
                      target_names = ['high', 'medium', 'low'],
                      title        = "Confusion Matrix")
```

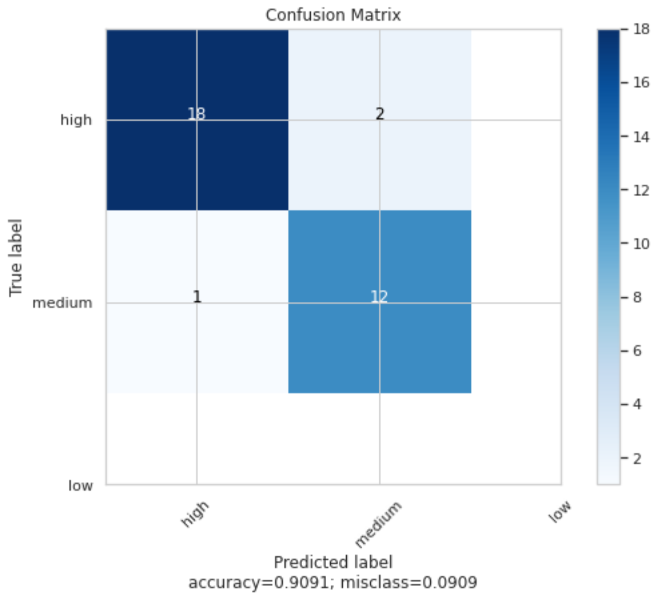


```
#NB
TP = 19
FP = 1
FN = 1
TN = 12

NBAcc = (TP + TN) / (TP + TN + FN + FP)
print('NB Testing Accuracy = ', NBAcc, '\n')
```

```
NB Testing Accuracy =  0.9393939393939394
```

```
plot_confusion_matrix(cm           = np.array([[ 19,  1],
                                               [ 1,  12  ]]),
                      normalize    = False,
                      target_names = ['high', 'medium', 'low'],
                      title        = "Confusion Matrix")
```

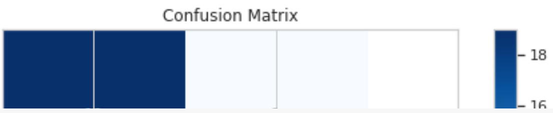


```
#DT
TP = 16
FP = 4
FN = 1
TN = 12

DTAcc = (TP + TN) / (TP + TN + FN + FP)
print('DT Testing Accuracy = ', DTAcc, '\n')
```

```
DT Testing Accuracy =  0.8484848484848485
```

```
plot_confusion_matrix(cm           = np.array([[ 16,  4],
                                               [  1,  12,  ]]),
                      normalize    = False,
                      target_names = ['high', 'medium', 'low'],
                      title        = "Confusion Matrix")
```

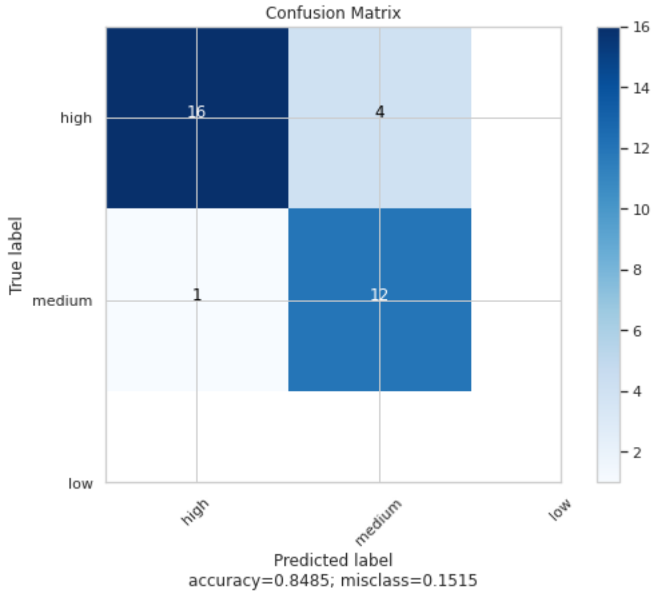


```
#DNN
TP = 18
FP = 2
FN = 1
TN = 12

DNNAcc = (TP + TN) / (TP + TN + FN + FP)
print('DNN Testing Accuracy = ', DNNAcc, '\n')
```

```
DNN Testing Accuracy =  0.9090909090909091
```

```
plot_confusion_matrix(cm           = np.array([[ 18,  2],
                                               [  1,  12,  ]]),
                      normalize    = False,
                      target_names = ['high', 'medium', 'low'],
                      title        = "Confusion Matrix")
```

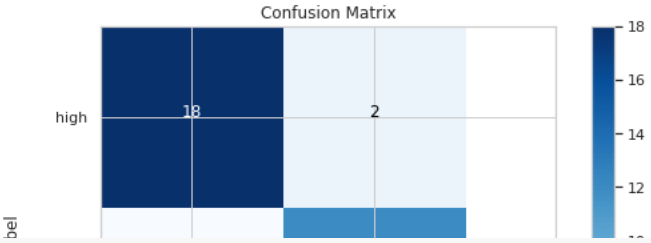


```
#RF
TP = 19
FP = 1
FN = 1
TN = 12

RFAcc = (TP + TN) / (TP + TN + FN + FP)
print('RF Testing Accuracy = ', RFAcc, '\n')
```

```
RF Testing Accuracy =  0.9393939393939394
```

```
plot_confusion_matrix(cm           = np.array([[ 19,  1],
                                               [  1,  12  ]]),
                      normalize    = False,
                      target_names = ['high', 'medium', 'low'],
                      title        = "Confusion Matrix")
```

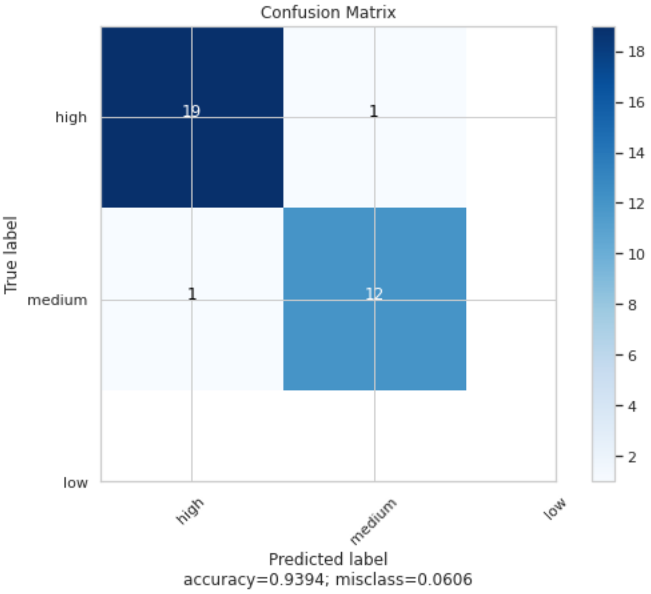


```
#KNN
TP = 18
FP = 2
FN = 4
TN = 9

KNNAcc = (TP + TN) / (TP + TN + FN + FP)
print('KNN Testing Accuracy = ', KNNAcc, '\n')
```

```
KNN Testing Accuracy =  0.8181818181818182
```

```
plot_confusion_matrix(cm           = np.array([[ 18,  2],
                                               [  4,  9  ]]),
                      normalize    = False,
                      target_names = ['high', 'medium', 'low'],
                      title        = "Confusion Matrix")
```

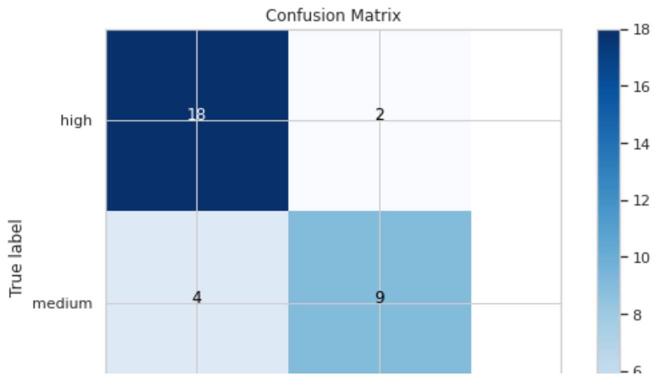

Print AUROC scores

```
print('SVMs: AUROC = %.3f' % (svm_auc))
print('Decsion Tree: AUROC = %.3f' % (tree_auc))
print('Random Forest: AUROC = %.3f' % (rf_auc))
print('Naive Bayes: AUROC = %.3f' % (nb_auc))
print('K-Nearest Neighbour: AUROC = %.3f' % (knn_auc))
print('DNN: AUROC = %.3f' % (dnn_auc))
```

```
SVMs: AUROC = 0.912
Decsion Tree: AUROC = 0.823
Random Forest: AUROC = 0.958
Naive Bayes: AUROC = 0.946
K-Nearest Neighbour: AUROC = 0.796
DNN: AUROC = 0.947
```

Plot the ROC curve

```
import matplotlib.pyplot as plt

svm_fpr = [1, 0.1, 0]
svm_tpr = [1, 0.923077, 0]

nb_fpr = [1, 0.95, 0.9, 0.85, 0.8, 0.75, 0.7, 0.65, 0.6, 0.55, 0.55, 0.5, 0.45, 0.4, 0.35, 0.3, 0.25, 0.2, 0.15, 0.1, 0.05, 0, 0]
nb_tpr = [1, 1, 1, 1, 1, 1, 1, 1, 1, 1, 0.923077, 0.923077, 0.923077, 0.923077, 0.923077, 0.923077, 0.923077, 0.923077, 0.923077, 0.92307

tree_fpr = [1, 0.2, 0.1, 0]
tree_tpr = [1, 0.923077, 0.076923, 0]

dnn_fpr = [1, 0.95, 0.9, 0.85, 0.8, 0.75, 0.7, 0.7, 0.65, 0.6, 0.55, 0.5, 0.45, 0.4, 0.35, 0.3, 0.25, 0.2, 0.15, 0.1, 0.05, 0, 0, 0, 0,
dnn_tpr = [1, 1, 1, 1, 1, 1, 1, 0.923077, 0.923077, 0.923077, 0.923077, 0.923077, 0.923077, 0.923077, 0.923077, 0.923077, 0.923077, 0.923
0.923077, 0.923077, 0.846154, 0.769231, 0.692308, 0.615385, 0.538462, 0.461538, 0.384615, 0.307692, 0.230769, 0.153846, 0.076923, 0]

rf_fpr = [1, 0.95, 0.9, 0.85, 0.75, 0.65, 0.55, 0.55, 0.45, 0.4, 0.35, 0.3, 0.25, 0.2, 0.15, 0.1, 0.05, 0, 0, 0, 0, 0, 0, 0, 0, 0, 0, 0]
rf_tpr = [1, 1, 1, 1, 1, 1, 1, 0.923077, 0.923077, 0.923077, 0.923077, 0.923077, 0.923077, 0.923077, 0.923077, 0.923077, 0.923077, 0.923
0.615385, 0.538462, 0.461538, 0.384615, 0.230769, 0.153846, 0.076923, 0]

knn_fpr = [1, 0.1, 0]
knn_tpr = [1, 0.692308, 0]
```

```
fig = plt.figure(figsize = (10, 10))

plt.plot(svm_fpr, svm_tpr, marker='.', label='SVM (AUROC = %0.3f)' % svm_auc)
plt.plot(nb_fpr, nb_tpr, marker='.', label='Naive Bayes (AUROC = %0.3f)' % nb_auc)
plt.plot(tree_fpr, tree_tpr, marker='.', label='Decision Tree (AUROC = %0.3f)' % tree_auc)
plt.plot(dnn_fpr, dnn_tpr, marker='.', label='Neaurla Networks (AUROC = %0.3f)' % dnn_auc)
plt.plot(rf_fpr, rf_tpr, marker='.', label='Random Forest (AUROC = %0.3f)' % rf_auc)
plt.plot(knn_fpr, knn_tpr, marker='.', label='K-Nearest Neighbour (AUROC = %0.3f)' % knn_auc)



# Title
plt.title('ROC Plot')
# Axis labels
plt.xlabel('False Positive Rate')
plt.ylabel('True Positive Rate')
```

```
# Show legend
plt.legend() #
# Show plot
plt.show()
```

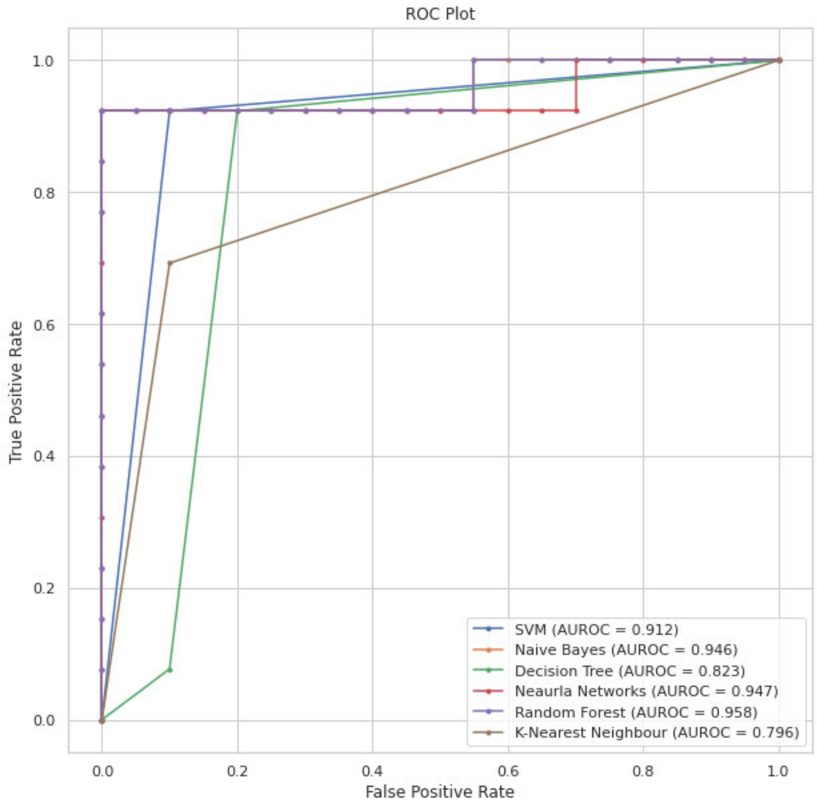

Class distribution

```
y.value_counts()
```

```
tissue: Tumor               56
tissue: adjacent nontumor   55
Name: CLASS, dtype: int64
```

```
# Show pie plot (Approach 1)
y.value_counts().plot.pie(autopct='%.2f')
```

```
<matplotlib.axes._subplots.AxesSubplot at 0x7f3b1885b350>
```

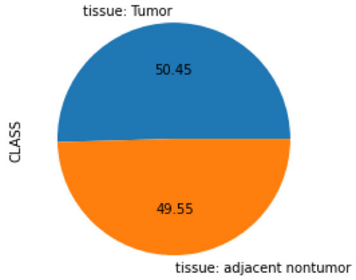

*Random Undersampling *

'not minority' = resample all classes but the minority class

```
from imblearn.under_sampling import RandomUnderSampler

rus = RandomUnderSampler(sampling_strategy=1) # Numerical value
# rus = RandomUnderSampler(sampling_strategy="not minority") # String
X_res, y_res = rus.fit_resample(X, y)
```

```
ax = y_res.value_counts().plot.pie(autopct='%.2f')
_ = ax.set_title("Under-sampling")
```

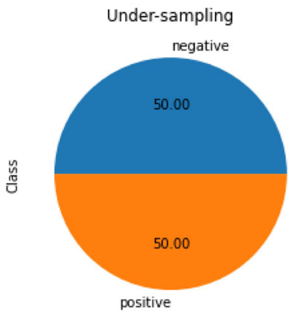


```
# Class distribution
y_res.value_counts()
```

```
negative    22
positive    22
Name: Class, dtype: int64
```
